# Cyclic diGMP Regulates Production of Sortase Substrates of *Clostridium difficile* and Their Surface Exposure through ZmpI Protease-mediated Cleavage*[Fn FN2]

**DOI:** 10.1074/jbc.M115.665091

**Published:** 2015-08-17

**Authors:** Johann Peltier, Helen A. Shaw, Edward C. Couchman, Lisa F. Dawson, Lu Yu, Jyoti S. Choudhary, Volkhard Kaever, Brendan W. Wren, Neil F. Fairweather

**Affiliations:** From the ‡ Department of Life Sciences, Center for Molecular Bacteriology and Infection, Imperial College London, London SW7 2AZ, United Kingdom,; the § Department of Pathogen Molecular Biology, London School of Hygiene and Tropical Medicine, London WC1E 7HT, United Kingdom,; the ¶ Wellcome Trust Sanger Institute, Hinxton CB10 1SA, United Kingdom, and; the ‖ Research Core Unit Metabolomics, Hannover Medical School, Hannover D-30625, Germany

**Keywords:** bacterial pathogenesis, cell surface protein, cyclic diGMP (c-diGMP), gene regulation, proteinase, Clostridium difficile, cell wall anchoring, sortase

## Abstract

In Gram-positive pathogens, surface proteins may be covalently anchored to the bacterial peptidoglycan by sortase, a cysteine transpeptidase enzyme. In contrast to other Gram-positive bacteria, only one single sortase enzyme, SrtB, is conserved between strains of *Clostridium difficile*. Sortase-mediated peptidase activity has been reported *in vitro,* and seven potential substrates have been identified. Here, we demonstrate the functionality of sortase in *C. difficile*. We identify two sortase-anchored proteins, the putative adhesins CD2831 and CD3246, and determine the cell wall anchor structure of CD2831. The C-terminal PPKTG sorting motif of CD2831 is cleaved between the threonine and glycine residues, and the carboxyl group of threonine is amide-linked to the side chain amino group of diaminopimelic acid within the peptidoglycan peptide stem. We show that CD2831 protein levels are elevated in the presence of high intracellular cyclic diGMP (c-diGMP) concentrations, in agreement with the control of CD2831 expression by a c-diGMP-dependent type II riboswitch. Low c-diGMP levels induce the release of CD2831 and presumably CD3246 from the surface of cells. This regulation is mediated by proteolytic cleavage of CD2831 and CD3246 by the zinc metalloprotease ZmpI, whose expression is controlled by a type I c-diGMP riboswitch. These data reveal a novel regulatory mechanism for expression of two sortase substrates by the secondary messenger c-diGMP, on which surface anchoring is dependent.

## Teaser

**Background:** Bacteria use various mechanisms to anchor their surface proteins, including a sortase enzyme.

**Results:** Covalent anchoring of proteins to the peptidoglycan in *Clostridium difficile* and its regulation by cyclic diGMP and protease activity are demonstrated.

**Conclusion:** A novel regulatory mechanism of cell wall protein anchoring is found.

**Significance:** Elucidating how proteins are anchored may shed light on control of bacterial colonization *in vivo*.

## Introduction

*Clostridium difficile*, a Gram-positive spore-forming bacterium, is the major cause of intestinal disease associated with antibiotic therapy (1). *C. difficile* infection is a serious risk within health care environments but is increasingly recognized as a community-associated disease (2, 3). Outbreaks leading to increased morbidity and mortality have been associated with highly virulent strains such as BI/NAP1/027 (4). *C. difficile* infection can result in a spectrum of symptoms, ranging from mild diarrhea to life-threatening pseudomembranous colitis and colon perforation (5). The major virulence factors mediating these symptoms are the toxins (TcdA and TcdB) that alter the actin cytoskeleton of intestinal epithelial cells through glycosylation of Rho family proteins, leading to a severe inflammatory response (6). Transmission of *C. difficile* is mediated by spores, which persist in the environment and are difficult to eradicate (7). *C. difficile* takes advantage of the disturbance of the normal colonic microbiota following antibiotic treatment to colonize the gastrointestinal tract (8). However, little is known about the mechanisms of gastrointestinal colonization, which could provide the basis to develop an effective treatment to reduce this problematic pathogen.

Bacterial surface structures such as pili and surface proteins can function as adhesins that enable pathogens to adhere to sites of infection (9). In Gram-positive bacteria, these virulence factors are often covalently attached to the peptidoglycan by a universal mechanism requiring membrane-associated enzymes termed sortases (10). Based on their primary sequences, sortase enzymes are currently grouped into six classes whose members have been shown to recognize different sorting motifs and to have distinct functions (11). The best studied is the class A sortase (SrtA), characterized in *Staphylococcus aureus* and other Gram-positive species and responsible for the anchoring of a large number of proteins to the cell wall (12). All classes of sortase catalyze a transpeptidation reaction involving cleavage at a specific motif in the C-terminal sorting signal of a sortase substrate, a surface-localized protein. In the case of class A sortases, this motif is “LP*X*TG,” with the exact motif varying between classes of sortase and species.

The sorting signal encompasses the LP*X*TG motif followed by a hydrophobic domain and a short positively charged tail (10). After synthesis in the bacterial cytoplasm, sortase substrate precursors are directed toward the Sec secretion system via their N-terminal secretion signal for translocation across the membrane. The hydrophobic domain and the positively charged tail ensure retention of the proteins in the membrane, allowing processing by sortase. Class A sortase cleaves the peptide bond between the threonine (Thr) and the glycine (Gly) group of the LP*X*TG motif and anchor the carboxyl group threonine to the peptidoglycan precursor lipid II before its incorporation to the mature peptidoglycan. In contrast, class B sortases recognize motifs with variations from the canonical LP*X*TG motif, and they have been proposed to anchor their substrates directly to the mature assembled peptidoglycan rather than to lipid II (13, 14).

In *C. difficile*, only one sortase gene (*CD2718*) is conserved in all sequenced strains. The sortase protein shows most sequence similarity to class B sortases and is annotated as SrtB (15). Recently, a structure for SrtB was determined, which is similar to other class B sortases (16). Bioinformatics approaches allowed the identification of seven putative sortase substrates in *C. difficile* 630, and the predicted sorting motif has been proposed to be (S/P)P*X*TG (17–19). Recently, functionality of SrtB has been demonstrated *in vitro*. Purified recombinant SrtB can cleave short (S/P)P*X*TG motif-containing peptides between the Thr and Gly residues and can covalently anchor the Thr residue to a nucleophile such as glycine or *meso-*diaminopimelic acid (mDAP)^5^ (17, 18). Additionally cell wall anchoring of the putative sortase substrate CD0386 is dependent on the activity of SrtB (16). However, there is no evidence that SrtB can covalently anchor proteins to the peptidoglycan of *C. difficile*.

Here, we demonstrate two proteins, CD2831 and CD3246, are anchored to the cell wall through sortase activity of SrtB that catalyzes covalent attachment of substrates to the mDAP of the peptidoglycan. CD2831 is a target for the metalloprotease ZmpI (CD2830) that cleaves its substrate at the C terminus, releasing it into the culture supernatant when levels of ZmpI are sufficient. CD2831 and ZmpI are inversely controlled by c-diGMP (20), and we show that increased synthesis of c-diGMP leads to cell wall anchoring of CD2831. This work reveals a novel association of sortase activity with c-diGMP-mediated regulation to control levels of cell wall anchoring and secretion of putative adhesion molecules.

## Experimental Procedures

### 

#### 

##### Growth Conditions

All strains used are listed in supplemental Table S1. *C. difficile* strains were routinely cultured on BHIS agar (brain heart infusion agar supplemented with 0.1% l-cysteine and 5 mg/ml yeast extract) or BHIS broth at 37 °C in an anaerobic environment (80% N_2_, 10% CO_2_, and 10% H_2_). When necessary, *C. difficile* culture media were supplemented with cycloserine (250 μg/ml), cefoxitin (25 μg/ml), or thiamphenicol (15 μg/ml). *C. difficile* minimal medium (21) supplemented with 5-fluorocytosine (50 μg/ml) was used to isolate double crossovers in *C. difficile*. Anhydrotetracycline (ATc, 5–100 ng/ml) was used for induction of the *P_tet_* promoter in the *C. difficile* expression vectors. *C. difficile* strains were generally grown to an absorbance at 600 nm (*A*_600 nm_) of 1–1.5. Unless specified otherwise, strains carrying pRPF185 derivatives were grown overnight in BHIS broth supplemented with 100 ng/ml ATc and subcultured to an *A*_600 nm_ of 0.1 in BHIS medium containing 100 ng/ml ATc. *Escherichia coli* NEB5α (New England Biolabs) was used for cloning and plasmid propagation. *E. coli* Rosetta (Novagen) was used to carry out protein expression and *E. coli* CA434 (HB101 carrying R702) was used for conjugation of plasmids into *C. difficile. E. coli* strains were cultured aerobically at 37 °C in LB broth or LB agar (MP Biomedicals) supplemented with chloramphenicol (15 μg/ml), carbenicillin (50 μg/ml), or kanamycin (50 μg/ml) where appropriate. For inducible protein expression, *E. coli* strains were grown in OverNight Express Instant TB broth (Novagen) supplemented with 10 ml/liter glycerol and the appropriate antibiotics.

##### Plasmid and Strain Construction

Plasmids and oligonucleotides are listed in supplemental Table S1 and were constructed using standard methods. For the expression of recombinant catalytic domain of endolysin CD27L (22), DNA encoding residues 1–179 of CD27L and an N-terminal His tag-encoding sequence were synthesized (GeneScript) and ligated between NdeI and XhoI sites in pET28a, yielding pHAS042.

For the expression of recombinant CD2831 (residues 32–290), primers NF1614 and NF1615 were used to PCR-amplify the corresponding DNA sequence. Purified PCR product was digested with NcoI and XhoI and ligated into the pET28a vector digested with the same enzymes, yielding pHAS027. For the expression of recombinant SrtB, *srtB* flanking primers NF1968 and NF1969 were used to PCR-amplify the *srtB* coding sequence deleted in the N-terminal hydrophobic domain. Purified PCR product was digested with NcoI and XhoI and ligated into the pET28a vector digested with the same enzymes, yielding pJKP001. All pET28a-derived plasmids were initially transformed into *E. coli* strain NEB5α (New England Biolabs), and sequences of all inserts were verified by sequencing. Plasmids were then transformed into *E. coli* strain Rosetta for protein expression and purification.

For expression of CD2831 in *C. difficile*, *CD2831* flanking primers NF2501 and NF2502 were used to PCR-amplify the *CD2831* coding sequence and its preceding RBS. For the expression of CD1420/DccA, *dccA* flanking primers NF2126/NF2199 were used to PCR-amplify the *dccA*-coding sequence and its preceding RBS and to add a C-terminal His tag. Purified PCR products were digested with SacI and BamHI and ligated into pRFP144, under control of the constitutive *P_cwp2_* promoter, or into pRFP185, under the control of the ATc-inducible *P_tet_* promoter (23), and digested with the same enzymes.

To introduce an arginyl-hemagglutinin (R-HA) epitope tag into CD2831, plasmid pJKP041 (carries *P_tet_-CD2831* for inducible CD2831 expression) was modified by inverse PCR with oligonucleotides NF2958 and NF2959, containing an appended sequence encoding for the arginyl-HA tag, yielding pJKP095. The arginyl-hemagglutinin tag was introduced five residues upstream of CD2831 PPKTG-sorting motif.

For the expression of CD3246_HA_, *CD3246*-flanking primers NF2860 and NF2861 were first used to PCR-amplify the *CD3246*-coding sequence. These two primers were designed using NEB Builder (New England Biolabs) so that DNA fragments to be fused contain an overlapping sequence with adjacent fragments. Because *CD3246* lacks a complete RBS, the RBS of *CD3392*, another putative sortase substrate, was added upstream from the *CD3246*-coding sequence. To achieve that, the *CD3392*-coding sequence and it preceding RBS were amplified by PCR with oligonucleotides NF2378 and NF2379, and purified PCR products were digested with SacI and BamHI and ligated into pRFP185, under the control of the ATc-inducible *P_tet_* promoter, and digested with the same enzymes. Inverse PCR was then carried out on the resulting plasmid using primers NF2451 and NF2858 to delete the entire *CD3392*-coding sequence but keep its RBS. Resulting plasmid was then assembled with previously amplified *CD3246*-coding sequence using the Gibson Assembly Master Mix (New England Biolabs). Resulting plasmid was then modified by inverse PCR with primers pair NF2726/NF2727 to add an HA tag-encoding sequence, yielding pJKP070. The HA tag was added five amino acids downstream from the CD3246 signal peptide.

Plasmids for protein expression in *C. difficile* were initially transformed into *E. coli* strain NEB5α (New England Biolabs), and sequences of all inserts were verified by sequencing. Plasmids were then transformed into *E. coli* CA434 and transferred by conjugation into the appropriate *C. difficile* strains.

The knock-out and knock-in strains generated in this study were created using the *codA*-mediated allele exchange method as described previously (24), with some modifications. For deletion of the *srtB* gene, a 1.85-kb DNA fragment comprising the gene to be deleted was amplified by PCR from 630 genomic DNAs using primers NF2155/NF2156. Purified PCR product was digested with EcoRI and BamHI and ligated into pUC19. Recombinant plasmid was purified and used as a template for inverse PCR with oligonucleotides NF2157/NF2158. This step introduced a central deletion of 586 bp for *srtB* gene. After purification, PCR product was ligated, and the resulting plasmid was used for PCR to amplify the entire sequence, which was then cloned into the PmeI site of pMTL-SC7215 vector, yielding pJKP019.

To construct *C. difficile P_tet_-srtB* with *srtB* genomic expression under the control of the *P_tet_*-inducible promoter, a 693-bp fragment upstream from the RBS of *srtB* and a 700-bp fragment encompassing the first 678 bases of *srtB* and its preceding RBS were amplified from 630 genomic DNAs using primers NF2254/NF2266 and NF2258/NF2259. Another 829-bp fragment comprising the entire *P_tet_* promoter and its preceding terminator was amplified from the pRFP185 plasmid (23) using primers NF2257/NF2267. All these primers were designed using NEB Builder (New England Biolabs) so that DNA fragments to be fused contain overlapping sequence with adjacent fragments. Purified PCR products were then ligated altogether into the PmeI site of pMTL-SC7215 vector using the Gibson Assembly Master Mix so that the *P_tet_* promoter-encompassing fragment was surrounded by the other PCR fragments, yielding pJKP029.

To complement the *srtB* mutant, the *srtB* gene was restored to its native locus in Δ*srtB.* A 1.8-kb DNA fragment encompassing the first 664 bp of *srtB* and its upstream region and a 1.3-kb DNA fragment encompassing the last 28 bp of *srtB* and its downstream region were amplified from 630 genomic DNA using primers NF2966/NF2967 and NF2968/2969. These primers were designed using NEB Builder (New England Biolabs), so that DNA fragments to be fused contain overlapping sequence with adjacent fragments. Primer NF2968 also introduced a codon substitution, so that the original TAA stop codon was replaced with a synonymous TAG codon. Purified PCR products were then directly ligated into the PmeI site of pMTL-SC7215 vector using the Gibson Assembly Master Mix, yielding pJKP096. This plasmid had a restored copy of the SrtB-encoding gene, the silent stop codon substitution making the complemented strain easily distinguishable from the wild-type strain.

For deletion of the ZmpI-encoding gene, ∼1.2-kb fragments up- and downstream of the sequence to be altered were amplified from 630 genomic DNA using primers NF2922/NF2923 and NF2924/NF2925. These primers were designed using NEB Builder (New England Biolabs) so that DNA fragments to be fused contain overlapping sequences with adjacent fragments. Purified PCR products were then directly ligated into the PmeI site of pMTL-SC7215 vector using the Gibson Assembly Master Mix, yielding pJKP088.

All pMTL-SC7215-derived plasmids were initially transformed into *E. coli* strain NEB5α (New England Biolabs), and sequences of all inserts were verified by sequencing. Plasmids were then transformed into *E. coli* CA434 and transferred by conjugation into the appropriate *C. difficile* strains. Transconjugants were selected on BHIS supplemented with cycloserine, cefoxitin, and thiamphenicol. A series of replica plating on thiamphenicol allowed faster growing single-crossover integrants to be isolated, and their purity was confirmed by PCR. Single crossover integrant colonies were then restreaked onto *C. difficile* minimal medium supplemented with fluorocytosine to isolate double crossovers. Fluorocytosine-resistant clones were restreaked onto BHIS supplemented with thiamphenicol to screen for plasmid loss. Thiamphenicol-sensitive clones were then tested by PCR for the presence of the expected deletions/insertions. All constructs were confirmed by sequencing.

##### Purification of CD27L, CD2831, and SrtB and Generation of Antibodies

Protein expression was induced in *E. coli* Rosetta by overnight cultivation in OverNight Express Instant TB medium. Recombinant proteins were purified by affinity chromatography in a HisTrap chelating HP column (GE Healthcare) on an AKTA liquid chromatography system (Pharmacia). Recombinant CD27L and recombinant SrtB were purified under native conditions, and recombinant CD2831 was purified under denaturating conditions and refolded on-column. Purified CD2831 and SrtB proteins were injected into BALB/c mice together with Freund's incomplete adjuvant to raise polyclonal antibodies.

##### Cell Lysis, Fractionation, and Protein Analysis

Whole cell lysates were prepared as described previously (23). Briefly, cultures of *C. difficile* were harvested by centrifugation at 5000 × *g* for 10 min at 4 °C, and the pellets were frozen at −20 °C. Bacteria were thawed, resuspended in PBS containing 0.12 μg/ml DNase I to an *A*_600 nm_ of 20, and incubated at 37 °C for 40 min. Culture supernatants were filtered with a 0.22-μm filter and precipitated on ice with 10% TCA for 30 min. Suspensions were harvested at 25,000 × *g* for 10 min at 4 °C, and the pellet was resuspended twice in ice-cold 90% acetone at 4 °C for 10 min. Finally, it was resuspended in PBS to an *A*_600 nm_ of 20. For cell fractionation, cultures of *C. difficile* were harvested by centrifugation at 5000 × *g* for 10 min at 4 °C and resuspended in phosphate/sucrose buffer (0.05 m HNa_2_PO_4_, pH 7.0, 0.5 m sucrose) to an *A*_600 nm_ of 20. Purified catalytic domain of endolysin CD27L was added to cell suspensions at 30 μg/ml, and samples were incubated at 37 °C for 1 h. Protoplasts were recovered by centrifugation at 6000 × *g* for 20 min at 4 °C. The supernatant corresponded to the cell wall (CW) fraction and the pellet to the protoplasts. Protoplasts were resuspended in phosphate buffer (0.05 m HNa_2_PO_4_, pH 7.0) containing 0.12 μg/ml DNase I at an *A*_600 nm_ of 20 and incubated at 37 °C for 45 min. Suspensions were harvested at 25,000 × *g* for 10 min at 4 °C to separate membrane and cytoplasm fractions. Membranes were finally resuspended in phosphate buffer at an *A*_600 nm_ of 20. For analysis by SDS-PAGE, an equal volume of 2× SDS sample buffer (25) was added to protein samples. SDS-PAGE and Western immunoblotting were carried out using standard methods.

##### Immunofluorescence Microscopy

*C. difficile* cells from liquid culture were washed with PBS and fixed in 8% formaldehyde in PBS, which was quenched using 20 mm NH_4_Cl for 15 min. The washed cell suspension was spotted onto a glass slide and allowed to dry. The bacteria were then incubated with 1:500 mouse anti-CD2831 and 1:500 rabbit anti-LMW-SLP and then washed and incubated with 1:200 anti-mouse rhodamine red and 1:200 anti-rabbit Cy2 (Jackson ImmunoResearch, West Grove, PA). Immunolabeled bacteria were visualized and photographed using a Nikon Eclipse E600 microscope fitted with a Retiga2000R Fast 1394 camera.

##### Purification of Cell Wall-anchored CD2831_R-HA_

*C. difficile* 630 Δ*zmpI* harboring pJKP095 was grown to an *A*_600 nm_ of 1.2 in 1 liter of BHIS liquid medium in the presence of the inducer ATc (100 ng/ml). Cells were harvested by centrifugation at 5000 × *g* for 10 min at 4 °C and resuspended in phosphate/sucrose buffer to an *A*_600 nm_ of 40. Purified catalytic domain of endolysin CD27L was added to the cell suspension at 30 μg/ml, and the sample was incubated at 37 °C for 1 h. Protoplasts were pelleted by centrifugation at 6000 × *g* for 20 min at 4 °C, and the supernatant containing the digested cell wall was collected. Cell wall extracts were concentrated using centrifugal filters (Amicon, Millipore) and resuspended twice in 15 ml of TBS buffer (50 mm Tris-Cl, pH 7.6, 150 mm NaCl). Cell wall extract was then concentrated to a final volume of 400 μl, and 80 μl of Pierce anti-HA-agarose (Thermo Fisher) slurry was added. After an overnight incubation at 4 °C with gentle shaking, resin was pelleted by centrifugation at 12,000 × *g* for 10 s, washed three times with TBS supplemented with 0.05% Tween 20, and then twice more with TBS. Cell wall-anchored CD2831_R-HA_ was eluted by addition of 50 mm NaOH to the resin. The eluate was collected by centrifugation at 12,000 × *g* for 10 s and was neutralized with 1 m Tris-HCl, pH 2.0.

##### Purification of Cell Wall-anchored C-terminal CD2831_R-HA_ Peptide

Fifty μl of purified CD2831_R-HA_ were digested with 1 μg of endoproteinase Arg-C (Roche Applied Science), according to the manufacturer's instructions. Arg-C cleaves at the C terminus of arginine residues. Cleaved peptides were then diluted in TBS, and the cell wall-anchored C-terminal CD2831_R-HA_ peptide was purified by immunoprecipitation using Pierce Anti-HA-agarose as above.

##### Peptide Identification by Liquid Chromatography-tandem Mass Spectrometry

The LC-MS/MS analysis was on an Ultimate 3000 RSLCnano System (Dionex) coupled to an LTQ OrbitrapVelos (Thermo Fisher) hybrid mass spectrometer equipped with a nanospray source. The peptides were first loaded and desalted on a PepMap C18 trap (0.1 mm inner diameter × 20 mm, 5 μm, Dionex) and then separated on a PepMap 75 μm inner diameter × 50 cm column with 3 μm particle size (Dionex) over a 60-min linear gradient of 4–36% CH_3_CN, 0.1% formic acid. The mass spectrometer was operated in the “top 10” data-dependent acquisition mode. The MS full scan was set at *m*/*z* 380–1600 with the resolution at 30,000 at *m*/*z* 400 and automatic gain control at 1 × 10^6^ with a maximum injection time at 200 ms. The 10 most abundant multiply charged precursor ions, with a minimal signal above 1000 counts, were dynamically selected for collision-induced dissociation fragmentation (MS/MS) in the linear trap quadrupole ion trap, which has the automatic gain control set at 5 × 10^3^ with the maximum injection time at 100 ms.

##### Measurement of Intracellular c-diGMP Levels

*C. difficile* strains were grown in 25 ml of BHIS containing thiamphenicol to an *A*_600 nm_ of 1.0. A 10-ml culture aliquot was harvested by centrifugation at 5000 × *g* for 10 min at 4 °C, washed, and freeze-dried to determine the dry weight for normalization purposes. Another 10-ml aliquot from the same culture was removed, and bacteria were collected by centrifugation at 5000 × *g* for 10 min at 4 °C. Nucleotides were extracted from the pellet with methanol/acetonitrile/milli-Q water (40:40:20) as described previously (26). The c-diGMP was detected and quantified by LC-MS/MS (26). The average intracellular concentration was determined by normalizing the c-diGMP measurements to the bacterial dry weight.

##### Sporulation Assay

Overnight cultures of *C. difficile* grown in BHIS medium supplemented with thiamphenicol and ATc, when suitable, were used to inoculate 10 ml of BHIS medium containing thiamphenicol. When necessary, ATc (100 ng/ml) was added every 24 h to maintain the protein induction. After 24 and 72 h of growth, 1 ml of culture was divided into two samples. To determine the total number of cfu, the first sample was serially diluted and plated on BHIS medium plus 0.1% taurocholate (Sigma). To determine the number of spores, the vegetative cells of the second sample were heat-killed by incubation for 25 min at 65 °C prior to plating on BHIS medium plus 0.1% taurocholate. The number of spores was subtracted from the total cell counts to give the vegetative cell numbers.

##### Aggregation Assay

Assessment of aggregation was performed as described previously with some modifications (27). Briefly, overnight cultures of *C. difficile* grown in BHIS medium supplemented with thiamphenicol and ATc, where appropriate, were used to inoculate 10 ml of fresh medium. After 8 h of growth, aggregates had settled to the bottom of the tube, and the suspension was mostly composed of free cells. Turbidity was measured at *A*_600 nm_ from the suspension of the unperturbed cultures. The cultures were then dispersed by vigorous vortexing, and the total turbidity was measured.

## Results

### 

#### 

##### Expression and Subcellular Localization of SrtB within C. difficile 630

**FIGURE 1.**
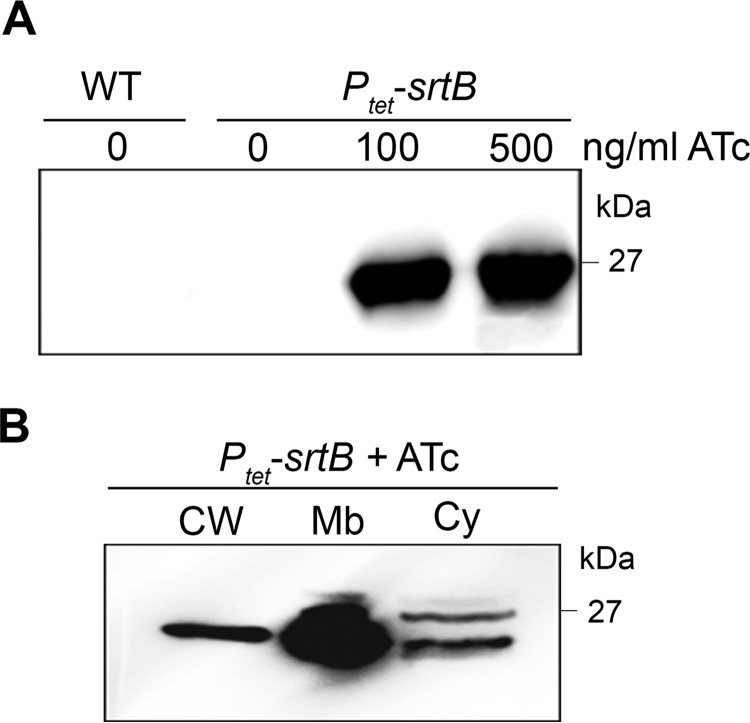
**Detection and localization of SrtB.** *A,* immunoblotting with anti-SrtB detected a major polypeptide of ∼25 kDa in whole cell extracts of the *P_tet_-srtB* strain grown in the presence of the inducer ATc (100 and 500 ng/ml) but not in extracts of wild-type strain 630 (*WT*) or not-induced *P_tet_-srtB* strain (0 ng/ml ATc). *B, P_tet_-srtB* culture induced with 100 ng/ml ATc was fractionated into cell wall (*CW*), membrane (*Mb*), and cytosolic (*Cy*) compartments and immunoblotted with anti-SrtB polyclonal antibodies. Proteins were separated on 12% SDS-polyacrylamide gels.

Expression levels of SrtB in *C. difficile* 630 were too low to detect efficiently by immunoblotting of whole cell extracts (Fig. 1*A*). We therefore used allele exchange to insert the inducible *P_tet_* promoter and its regulatory element into the chromosome between the native *srtB* promoter and the *srtB* gene, leading to the *P_tet_-srtB* strain. SrtB was detectable in whole cell extracts of the *P_tet_-srtB* strain with 100 or 500 ng/ml inducer ATc (Fig. 1*A*). No band was observed without induction of SrtB expression. The distribution of SrtB within cytosolic, membrane, and cell wall (peptidoglycan) fractions in the *P_tet_-srtB* strain induced with 100 ng/ml ATc was studied. As expected, SrtB was mainly found located in the membrane fraction (Fig. 1*B*).

##### CD2831 Is a Surface-exposed Protein Released by the ZmpI Protease

**FIGURE 2.**
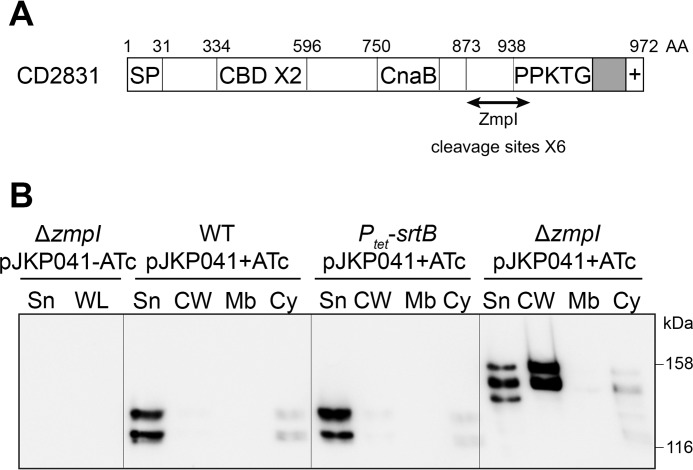
**ZmpI-mediated release of the cell wall-associated CD2831 protein into the culture supernatant.** *A,* schematic representation of CD2831. The relevant characteristics are a signal peptide (*SP*), two putative collagen-binding domains (*CBD*), a CnaB-like domain, and a C-terminal putative sorting signal comprising a PPKTG sorting motif, a hydrophobic domain (in *gray*), and a positively charged tail (+). *AA,* amino acids. *B,* supernatant (*Sn*), cell wall (*CW*), membrane (*Mb*), and cytosolic (*Cy*) compartment of wild type (*WT*), *P_tet_-srtB* (overexpresses the sortase in the presence of ATc), and *zmpI* mutant (Δ*zmpI*) strains, carrying pJKP041 (expresses CD2831 in the presence of ATc) and cultivated in the presence of 100 ng/ml ATc (+*ATc*), were analyzed by Western blotting with anti-CD2831 polyclonal antibodies. Supernatant and whole cell lysate (*WL*) fractions of Δ*zmpI* pJKP041 strain cultivated in the absence of ATc (−*ATc*) were also analyzed as negative controls.

Seven putative sortase substrates have been identified in *C. difficile* 630, but only four are conserved in the ribotype 027 strain R20291 (17). Their putative functions are as follows: CD0183 and CD2768, cell wall hydrolases; CD2537, a 5′-nucleotidase/phosphoesterase; and CD2831, a collagen-binding protein. CD2831 contains two collagen-binding domains and a CnaB-like domain (Fig. 2*A*). CnaB domains, first identified in *S. aureus* (28), are commonly found in cell-surface adhesins and are thought to form a stalk presenting the ligand-binding domain away from the bacterial cell surface (29). CD2831 is efficiently cleaved *in vitro* by the secreted zinc metalloprotease ZmpI (CD2830) (30, 31). Six different ZmpI target cleavage sites have been identified within the C terminus of CD2831 (Fig. 2*A*) and a proteomic analysis of the extracellular medium of *C. difficile* cultures revealed that CD2831 is at least partly secreted (30). To give further insight into the cellular localization of CD2831, immunoblotting was used to localize the protein in cellular fractions and culture supernatants of several strains. CD2831 was poorly detected in both the supernatant and in whole cell lysate of strain 630 (Fig. 2*B* and data not shown), suggesting that the normal levels of CD2831 are low. Therefore, the *CD2831* gene was cloned under control of the inducible *P_tet_* promoter on a plasmid, yielding pJKP041. Strain 630 (pJKP041) was grown in the presence of inducer, and the distribution of CD2831 in supernatant, cell wall, membrane, and cytoplasmic fractions was investigated. Two polypeptides were detected by Western blotting, observed mainly in the culture supernatant (Fig. 2*B*). Because SrtB expression was undetectable in the wild-type 630 strain, secretion of the putative sortase substrate CD2831 into the culture supernatant could be due to the absence of sortase, leading to the inability to anchor CD2831 to the cell surface. Therefore, pJKP041 was introduced into the *P_tet_-srtB* strain, and the resultant strain was grown in the presence of inducer. However, the subcellular distribution of CD2831 remained unchanged in this strain (Fig. 2*B*), demonstrating that the secretion of CD2831 into the culture supernatant is independent of SrtB.

To investigate whether the secretion of CD2831 is a result of its cleavage by the ZmpI protease, a *zmpI* deletion mutant was generated in strain 630. pJKP041 was then introduced in this strain, and subcellular localization of CD2831 in cells grown in the presence of inducer was investigated. Although a small portion of CD2831 was detected in the supernatant fraction, the majority of CD2831 was localized in the cell wall fraction (Fig. 2*B*). Interestingly, a clear difference was observed in the molecular weights of CD2831 depending on the production of the ZmpI protease. In the wild-type strain where ZmpI is produced, CD2831 migrates as two major polypeptides with sizes of ∼120 and 130 kDa. In contrast, polypeptides detected in the Δ*zmpI* strain migrate at larger masses, between 140 and 155 kDa. This is consistent with CD2831 being a substrate for the proteolytic activity of ZmpI. These data indicate that CD2831 can be associated with the cell wall and that ZmpI-mediated cleavage is essential for complete release of CD2831 from the cell surface.

**FIGURE 3.**
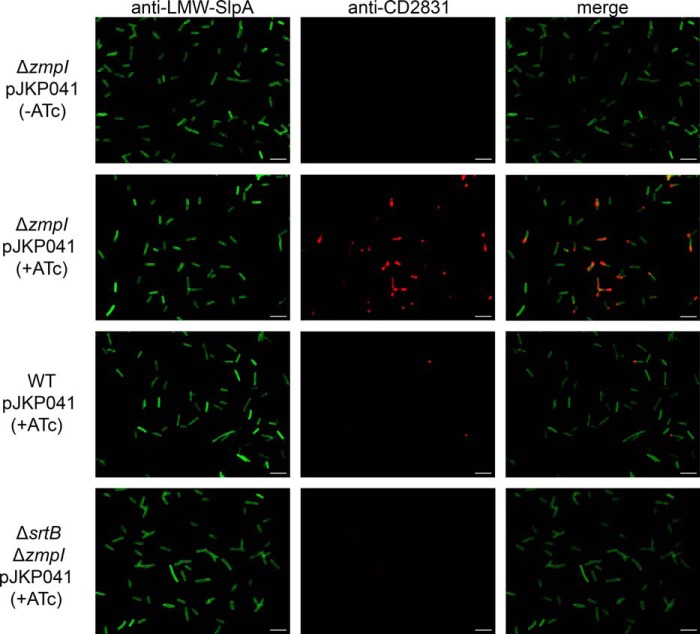
**Display of CD2831 on the clostridial cell surface.** The wild-type (*WT*), *zmpI* mutant (Δ*zmpI*), and *srtB*/*zmpI* double mutant (Δ*srtB*Δ*zmpI*) strains carrying pJKP041 (overexpresses CD2831 in the presence of ATc) were grown in BHIS broth in the presence of 100 ng/ml ATc (+*ATc*) and labeled with rabbit anti-LMW-SlpA (*green, left panels*) to label the surface of all bacteria and mouse anti-CD2831 (*red, middle panels*) to assess CD2831 localization. Overlays of those images are also showed (*merge, right panels*). Labeling was visualized using Cy2 anti-rabbit conjugate and rhodamine red-X anti-mouse conjugate. The Δ*zmpI* strain carrying pJKP041 and grown in the absence of the inducer (−*ATc*) was used as a negative control. *White bars* represent 10 μm.

To assess CD2831 localization at the individual cell level, immunofluorescence microscopy analysis was performed on intact bacteria. All bacteria were uniformly labeled with anti-LMW SLP, which detects the S-layer that completely coats *C. difficile* (Fig. 3). CD2831 could be detected at the surface of the induced Δ*zmpI* pJKP041 strain (Fig. 3), but the intensity of labeling by the anti-CD2831 antibodies appeared to be heterogeneous in the bacterial population. Interestingly, CD2831 labeling was not distributed along the entire bacterial cell but was concentrated mostly at one cell pole. Similar features have been previously reported for SrtB substrates in *Listeria monocytogenes* (see below) (32). Most cells from the induced wild-type pJKP041 culture were not stained with anti-CD2831, with the exception of polar staining in a few cells. This residual fluorescence was not observed in the uninduced Δ*zmpI* pJKP041 strain, indicating that a small fraction of CD2831 remains at the cell surface in the induced wild-type pJKP041 strain. It is likely that the level of ZmpI in the extracellular medium is insufficient to cleave efficiently the large amount of CD2831 protein produced under inducing conditions.

##### SrtB Anchors CD2831 to the Cell Wall

**FIGURE 4.**
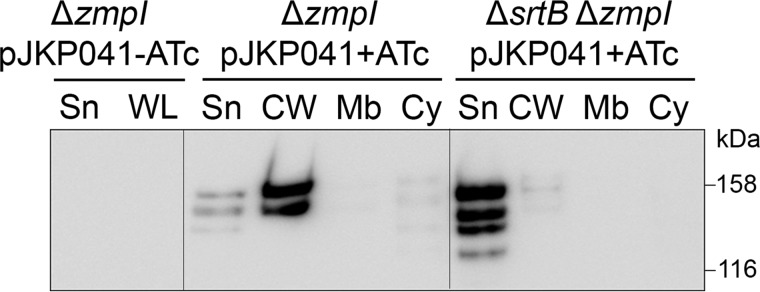
**Role of SrtB in anchoring CD2831 to the cell wall.** Supernatant (*Sn*), cell wall (*CW*), membrane (*Mb*), and cytosolic (*Cy*) compartment of *zmpI* mutant (Δ*zmpI*) and *srtB*/*zmpI* double mutant (Δ*srtB*Δ*zmpI*) strains, carrying pJKP041 (expresses CD2831 in the presence of ATc) and cultivated in the presence of 100 ng/ml ATc (+*ATc*) were analyzed by Western blotting with anti-CD2831 polyclonal antibodies. Supernatant and whole cell lysate (*WL*) fractions of Δ*zmpI* pJKP041 strain cultivated in the absence of ATc (−*ATc*) were also analyzed as negative controls.

The ZmpI cleavage sites within CD2831 are not randomly distributed along the protein sequence but are confined in the C-terminal extremity, directly upstream of the putative PPKTG sorting motif (30). Thus, it is possible that the CD2831 protein is anchored to the cell wall through a sorting reaction catalyzed by *SrtB* and subsequently secreted into the extracellular medium after its cleavage by the ZmpI protease. To test this hypothesis, the *srtB* gene in the Δ*zmpI* mutant strain was inactivated by deletion. pJKP041 was then introduced into the Δ*zmpI*Δ*srtB* double mutant, and the subcellular localization of CD2831 in this strain grown in the presence of inducer was then analyzed by immunoblotting and immunofluorescence microscopy. Cell wall extracts from the Δ*zmpI*Δ*srtB* mutant displayed undetectable levels of CD2831, whereas the protein was strongly detected in the supernatant fraction (Fig. 4). In agreement with these data, the anti-CD2831 antibodies did not surface-label the Δ*zmpI*Δ*srtB* mutant strain (Fig. 3), demonstrating that SrtB is required for anchoring of CD2831 to the cell wall.

##### Cell Wall Anchor Structure of Endolysin-solubilized CD2831_R-HA_ Tag

**FIGURE 5.**
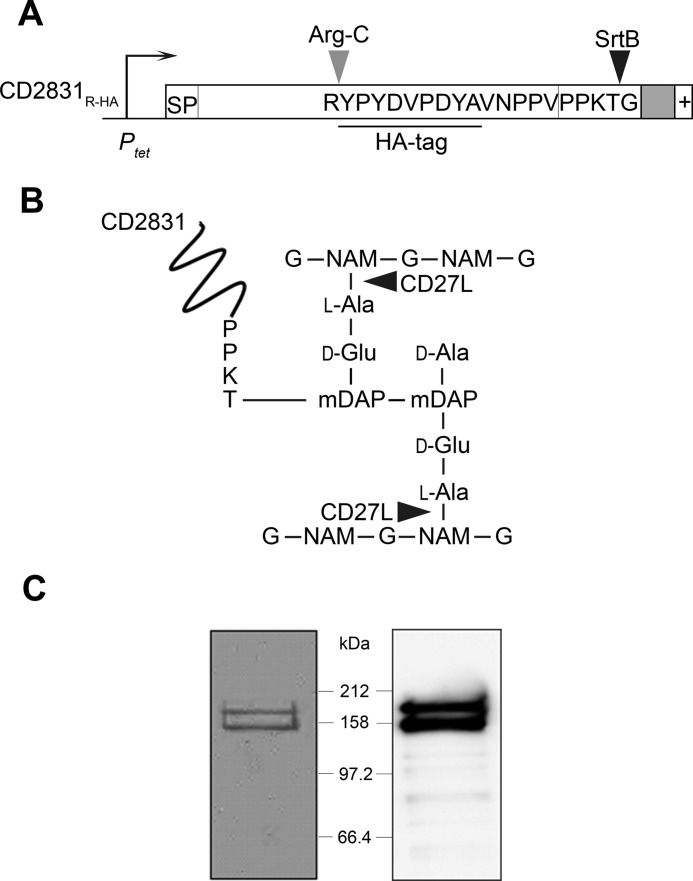
**Expression and purification of CD2831_R-HA_ from the cell wall of *C. difficile* Δ*zmpI*.** *A,* CD2831_R-HA_ construct under control of the inducible tetracycline promoter (*P_tet_*) containing signal peptide (*SP*), the PPKTG motif, the hydrophobic domain (in *gray*), and the positively charged tail (+). Cleavage sites of SrtB and ArgC are indicated. *B,* CD2831 is covalently linked to the peptidoglycan of *C. difficile*. Nonacetylated glucosamine residues (*G*) and unusual mDAP-mDAP cross-links generated by dl-transpeptidation are predominant in the peptidoglycan of *C. difficile* (34). Cleavage sites of the CD27L endolysin are indicated by *arrowheads. NAM*, *N*-acetylmuramic acid; *DAP*, diaminopimelic acid. *C,* cells from Δ*zmpI* strain expressing CD2831_R-HA_ were suspended in PS buffer and treated with the purified catalytic domain of the CD27L endolysin. Digested cell wall was isolated by centrifugation and subjected to immunoprecipitation using anti-HA. Purified polypeptides were separated on 10% SDS-polyacrylamide gels and Coomassie Blue-stained (*left panel*) or probed with anti-HA antibodies (*right panel*).

SrtB-dependent cell wall anchoring of CD2831 suggests its covalent anchoring to the peptidoglycan. To determine the cell wall anchor structure of CD2831, a C-terminally modified derivative (CD2831_R-HA_) was constructed and placed under the control of the inducible *P_tet_* promoter (pJKP095). CD2831_R-HA_ consists of CD2831 with an arginine residue and an HA tag inserted five amino acids upstream of the PPKTG sorting motif (Fig. 5*A*). pJKP095 was transferred into the Δ*zmpI* mutant, and upon induction with ATc, CD2831_R-HA_ could be detected in both the supernatant and the cell wall fraction by immunoblotting with anti-CD2831 or anti-HA antibodies, indicating that the subcellular localization of CD2831_R-HA_ is similar to that of CD2831 (data not shown).

**FIGURE 6.**
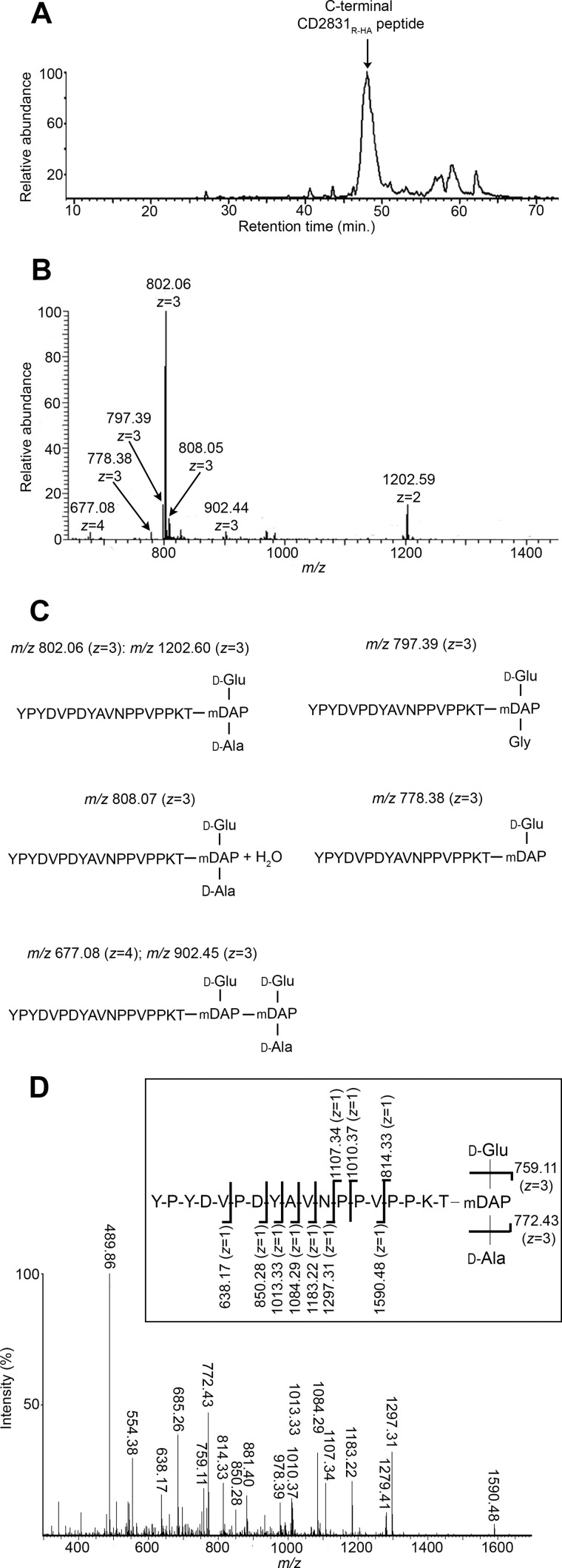
**Structure of CD27L-released CD2831_R-HA_ anchor peptides.** Reverse phase-HPLC elution profile (*A*) and mass spectrometry spectrum (*B*) of CD2831_R-HA_ anchor peptides. Purified CD2831_R-HA_ was cleaved with Arg-C to generate C-terminal CD2831_R-HA_ anchor peptides, which were further purified by a second round of anti-HA immunoprecipitation. Purified anchor peptides were analyzed by LC-MS. *C,* deduced structures and their calculated *m/z* value for the different species detected by mass spectrometry. *D,* MS/MS sequencing of the predominant ion at *m/z* 802.06 with *z* = 3. Inferred structure is represented. The indicated *m/z* values on the presented structure correspond to ions obtained by cleavage of one amide bond.

**FIGURE 7.**
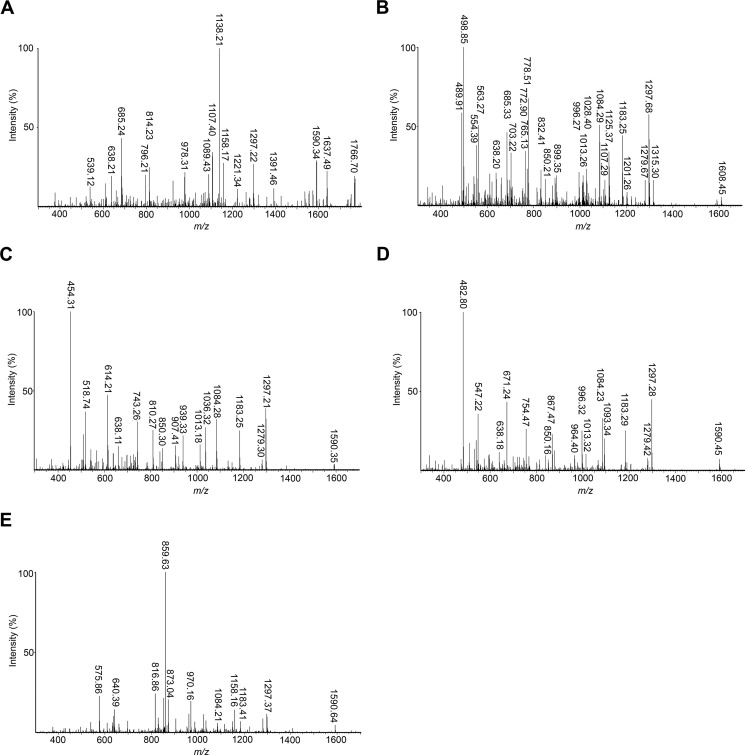
**MS/MS sequencing of the different ions corresponding to the CD2831_R-HA_ anchor peptides and identified by LC-MS in Fig. 6*B*.** MS/MS sequencing of the ions at *m/z* 1202.59 with *z* = 2 (*A*), *m/z* 808.05 with *z* = 3 (*B*), *m/z* 778.38 with *z* = 3 (*C*), *m/z* 797.39 with *z* = 3 (*D*), and *m/z* 902.44 with *z* = 3 (*E*).

The cell wall fraction of *C. difficile* expressing CD2831_R-HA_ was solubilized by digestion with CD27L, a *C. difficile* phage-endolysin predicted to have *N*-acetylmuramoyl-l-amidase activity (Fig. 5*B*) (22, 33). Cell wall-anchored CD2831_R-HA_ was then purified from this digest by immunoprecipitation with an anti-HA affinity resin (Fig. 5*C*). The enriched protein was subjected to digestion with the endopeptidase Arg-C, which cleaves at the C terminus of arginine residues, and C-terminal peptides harboring the HA tag were purified again by immunoprecipitation. The structures of the resulting eluted peptides, expected to be covalently anchored to peptidoglycan peptide side chains, were then analyzed by a combination of LC-MS and MS/MS. All peptides corresponding to CD2831 eluted as a single wide peak (Fig. 6*A*). The seven prominent ions contained in this peak were assigned based on their mass determination to structures in agreement with an HA-tagged C-terminal CD2831 peptide that terminates at the threonine residue of the PPKTG sorting motif and is covalently linked to the peptide side chain of peptidoglycan (Fig. 6, *B* and *C*). Among the different ion signals observed, the predominant ion at *m/z* 802.06 with *z* = 3 and the minor ion at *m/z* 1202.59 with *z* = 2 are proposed to have identical structures, consisting of the anchor peptide linked to cell wall tripeptide (d-Glu-(YPYDVPDYAVNPPVPPKT-)mDAP-d-Ala). Both ions were subjected to MS/MS analysis, and the main daughter ions were assigned to the corresponding peptide structure (Figs. 6*D* and 7*A* and Tables 1 and 2). In both cases, the fragmentation pattern was consistent with the predicted structure and thus confirmed that CD2831 is covalently anchored to the peptidoglycan. The alanine residue in the cell wall tripeptide was unambiguously found to be located at the C terminus of the peptide chain of the peptidoglycan rather than at its N terminus. Indeed, fragmentation of the parental ion at *m/z* 802.06 (*z* = 3) led to the generation of several daughter ions lacking the glutamate residue but not the alanine residue (fragments with an *m/z* 489.86, *z* = 2; *m/z* 685.26, *z* = 1; *m/z* 759.11, *z* = 1; *m/z* 881.40, *z* = 1; and *m/z* 978.39, *z* = 1) and to the generation of a daughter ion at *m/z* 772.43 (*z* = 3) corresponding to the loss of an alanine residue (−89.05 Da) from the C-terminal end of the peptide stem. Moreover, because either the glutamate or alanine residue could be individually lost, this result strongly suggests that the anchor peptide is linked to the mDAP residue of the peptide side chain of peptidoglycan. The minor ion at *m/z* 808.05 (*z* = 3) had a 17.95- Da mass difference compared with the predominant ion signal, suggesting the addition of a water molecule (Fig. 6, *B* and *C*). MS/MS fragmentation of this ion revealed that this additional mass could be easily lost as most of the daughter ions presented two different forms, with or without the extra 18 Da. Moreover, this mass could be lost either from the N-terminal extremity of the anchor peptide or from the peptidoglycan peptide stem (Fig. 7*B* and Table 3). Therefore, the ion at *m/z* 808.05 (*z* = 3) corresponds to the H^+^ + H_2_O adduct of the main structure identified above.

**TABLE 1:** **Proposed structures of fragment ions from MS/MS collision-induced dissociation of parental ion at *m/z* 802.06 (*z* = 3)** See Fig. 6*D.*

Ion formed	*m/z*		Proposed structure
	Calculated	Observed	
[M + 2H]^2+^	489.80	489.86	(PPVPPKT-)mDAP-d-Ala
[M + 2H]^2+^	554.36	554.38	d-Glu- (PPVPPKT-)mDAP-d-Ala
[M + H]^+^	638.28	638.17	YPYDV
[M + H]^+^	685.41	685.26	(PPKT-)mDAP-d-Ala
[M + 3H]^3+^	759.05	759.11	mDAP- (YPYDVPDYAVNPPVPPKT-)d-Ala
[M + 3H]^3+^	772.38	772.43	d-Glu- (YPYDVPDYAVNPPVPPKT-)mDAP
[M + H]^+^	814.45	814.33	d-Glu- (PPKT-)mDAP-d-Ala
[M + H]^+^	850.36	850.28	YPYDVPD
[M + H]^+^	881.53	881.40	(PVPPKT-)mDAP-d-Ala
[M + H]^+^	978.58	978.39	(PPVPPKT-)mDAP-d-Ala
[M + H]^+^	1010.57	1010.37	d-Glu- (PVPPKT-)mDAP-d-Ala
[M + H]^+^	1013.43	1013.33	YPYDVPDY
[M + H]^+^	1084.46	1084.29	YPYDVPDYA
[M + H]^+^	1107.62	1107.34	d-Glu- (PPVPPKT-)mDAP-d-Ala
[M + H]^+^	1183.53	1183.22	YPYDVPDYAV
[M + H]^+^	1279.67	1279.41	DYAVNPPVPPKT
[M + H]^+^	1297.57	1297.31	YPYDVPDYAVN
[M + H]^+^	1590.75	1590.48	YPYDVPDYAVNPPV

**TABLE 2:** **Proposed structures of fragment ions from MS/MS collision-induced dissociation of parental ion at *m/z* 1202.59 (*z* = 2)** See Fig. 7*A.*

Ion formed	*m/z*		Proposed structure
	Calculated	Observed	
[M + H]^+^	539.21	539.12	YPYD
[M + H]^+^	638.28	638.21	YPYDV
[M + H]^+^	685.41	685.24	(PPKT-)mDAP-d-Ala
[M + H]^+^	796.44	796.21	d-Glu- (PPKT-)mDAP-d-Ala − H_2_O
[M + H]^+^	814.45	814.23	d-Glu- (PPKT-)mDAP-d-Ala
[M + H]^+^	978.58	978.31	(PPVPPKT-)mDAP-d-Ala
[M + H]^+^	1089.61	1089.43	d-Glu- (PPVPPKT-)mDAP-d-Ala − H_2_O
[M + H]^+^	1107.62	1107.40	d-Glu- (PPVPPKT-)mDAP-d-Ala
[M + 2H]^2+^	1138.08	1138.21	(YPYDVPDYAVNPPVPPKT-)mDAP-d-Ala
[M + 2H]^2+^	1158.07	1158.17	d-Glu- (YPYDVPDYAVNPPVPPKT-)mDAP
[M + H]^+^	1221.67	1221.34	d-Glu- (NPPVPPKT-)mDAP-d-Ala
[M + H]^+^	1297.57	1297.22	YPYDVPDYAVN
[M + H]^+^	1391.77	1391.46	d-Glu- (AVNPPVPPKT-)mDAP-d-Ala
[M + H]^+^	1590.75	1590.34	YPYDVPDYAVNPPV
[M + H]^+^	1637.87	1637.49	(PDYAVNPPVPPKT-)mDAP-d-Ala
[M + H]^+^	1766.92	1766.70	d-Glu- (PDYAVNPPVPPKT-)mDAP-d-Ala

**TABLE 3:** **Proposed structures of fragment ions from MS/MS collision-induced dissociation of parental ion at *m/z* 808.05 (*z* = 3)** See Fig. 7*B.*

Ion formed	*m/z*		Proposed structure
	Calculated	Observed	
[M + 2H]^2+^	489.80	489.91	(PPVPPKT-)mDAP-d-Ala
[M + 2H + H_2_O]^2+^	498.80	498.85	(PPVPPKT-)mDAP-d-Ala
[M + 2H]^2+^	554.36	554.39	d-Glu- (PPVPPKT-)mDAP-d-Ala
[M + 2H + H_2_O]^2+^	563.37	563.27	d-Glu- (PPVPPKT-)mDAP-d-Ala
[M + H]^+^	638.28	638.20	YPYDV
[M + H]^+^	685.41	685.33	(PPKT-)mDAP-d-Ala
[M + H + H_2_O]^+^	703.42	703.22	(PPKT-)mDAP-d-Ala
[M + 3H + H_2_O]^3+^	765.05	765.13	(YPYDVPDYAVNPPVPPKT-)mDAP-d-Ala
[M + 3H]^3+^	772.38	772.90	d-Glu- (YPYDVPDYAVNPPVPPKT-)mDAP
[M + 3H + H_2_O]^3+^	778.38	778.51	d-Glu- (YPYDVPDYAVNPPVPPKT-)mDAP
[M + H + H_2_O]^+^	832.46	832.41	d-Glu- (PPKT-)mDAP-d-Ala
[M + H]^+^	850.36	850.21	YPYDVPD
[M + H + H_2_O]^+^	899.54	899.35	(PVPPKT-)mDAP-d-Ala
[M + H + H_2_O]^+^	996.59	996.27	(PPVPPKT-)mDAP-d-Ala
[M + H]^+^	1013.43	1013.26	YPYDVPDY
[M + H + H_2_O]^+^	1028.58	1028.40	d-Glu-mDAP- (PVPPKT-)d-Ala
[M + H]^+^	1084.46	1084.29	YPYDVPDYA
[M + H]^+^	1107.62	1107.29	d-Glu- (PPVPPKT-)mDAP-d-Ala
[M + H + H_2_O]^+^	1125.63	1125.37	d-Glu- (PPVPPKT-)mDAP-d-Ala
[M + H]^+^	1183.53	1183.25	YPYDVPDYAV
[M + H + H_2_O]^+^	1201.54	1201.26	YPYDVPDYAV
[M + H]^+^	1279.67	1280.43	DYAVNPPVPPKT
[M + _2_O]^+^	1297.68	1297.26	DYAVNPPVPPKT
[M + H + H_2_O]^+^	1315.58	1315.30	YPYDVPDYAVN
[M + H + H_2_O]^+^	1608.76	1608.45	YPYDVPDYAVNPPV

The minor ion signal at *m/z* 778.38 with *z* = 3 had a 70.99-Da mass difference compared with the predominant ion signal, indicating the lack of an alanine residue (Fig. 6, *B* and *C*). Therefore, the corresponding structure was deduced to be d-Glu-(YPYDVPDYAVNPPVPPKT-)mDAP, which was confirmed by MS/MS analysis. This MS/MS fragmentation generated an ion fragment with an *m/z* 614.21 (*z* = 1), corresponding to the C-terminal PPKT anchor peptide linked to the mDAP of the peptidoglycan peptide stem (Fig. 7*C* and Table 4). This result confirms that the carboxyl group of threonine is linked to the free amino group of the mDAP of the peptidoglycan peptide side chain.

**TABLE 4:** **Proposed structures of fragment ions from MS/MS collision-induced dissociation of parental ion at *m/z* 778.38 (*z* = 3)** See Fig. 7*C.*

Ion formed	*m/z*		Proposed structure
	Calculated	Observed	
[M + 2H]^2+^	454.28	454.31	(PPVPPKT-)mDAP
[M + 2H]^2+^	518.84	518.74	d-Glu- (PPVPPKT-)mDAP
[M + H]^+^	614.37	614.21	(PPKT-)mDAP
[M + H]^+^	638.28	638.11	YPYDV
[M + H]^+^	743.41	743.26	d-Glu- (PPKT-)mDAP
[M + H]^+^	810.49	810.27	(PVPPKT-)mDAP
[M + H]^+^	850.36	850.30	YPYDVPD
[M + H]^+^	907.54	907.41	(PPVPPKT-)mDAP
[M + H]^+^	939.53	939.33	d-Glu- (PVPPKT-)mDAP
[M + H]^+^	1013.43	1013.18	YPYDVPDY
[M + H]^+^	1036.59	1036.32	d-Glu- (PPVPPKT-)mDAP
[M + H]^+^	1084.46	1084.28	YPYDVPDYA
[M + H]^+^	1183.53	1183.25	YPYDVPDYAV
[M + H]^+^	1279.67	1279.30	DYAVNPPVPPKT
[M + H]^+^	1297.57	1297.21	YPYDVPDYAVN
[M + H]^+^	1590.49	1590.35	YPYDVPDYAVNPPV

The signal at *m/z* 797.39 (*z* = 3) had a 14-Da mass difference compared with the predominant ion (Fig. 6*B*), and MS/MS fragmentation of this ion showed that the C-terminal d-Ala of the cell wall peptide had been replaced by a glycine (Fig. 7*D* and Table 5). This feature is in agreement with the established structure of cell wall peptidoglycan of vegetative *C. difficile* cells, as a small proportion of muropeptides harboring glycine at position 4 of the peptide stem instead of d-alanine was reported (34).

**TABLE 5:** **Proposed structures of fragment ions from MS/MS collision-induced dissociation of parental ion at *m/z* 797.39 (*z* = 3)** See Fig. 7*D.*

Ion formed	*m/z*		Proposed structure
	Calculated	Observed	
[M + 2H]^2+^	482.78	482.80	(PPVPPKT-)mDAP-Gly
[M + 2H]^2+^	547.30	547.22	d-Glu- (PPVPPKT-)mDAP-Gly
[M + H]^+^	638.28	638.18	YPYDV
[M + H]^+^	671.39	671.24	(PPKT-)mDAP-Gly
[M + 3H]^3+^	754.38	754.47	(YPYDVPDYAVNPPVPPKT-) mDAP-Gly
[M + H]^+^	850.36	850.16	YPYDVPD
[M + H]^+^	867.51	867.47	(PVPPKT-)mDAP-Gly
[M + H]^+^	964.56	964.40	(PPVPPKT-)mDAP-Gly
[M + H]^+^	996.55	996.32	d-Glu- (PVPPKT-)mDAP-Gly
[M + H]^+^	1013.43	1013.32	YPYDVPDY
[M + H]^+^	1084.46	1084.23	YPYDVPDYA
[M + H]^+^	1093.61	1093.34	d-Glu- (PPVPPKT-)mDAP-Gly
[M + H]^+^	1183.53	1183.29	YPYDVPDYAV
[M + H]^+^	1279.67	1279.42	DYAVNPPVPPKT
[M + H]^+^	1297.57	1297.28	YPYDVPDYAVN
[M + H]^+^	1590.75	1590.45	YPYDVPDYAVNPPV

The minor ion signals at *m/z* 677.08 (*z* = 4) and 902.44 (*z* = 3) had an extra mass of 301.14 Da compared with the predominant ion, which matched with the addition of the dipeptide side chain d-Glu-mDAP (Fig. 6, *B* and *C*). To examine the validity of our prediction, the parental ion at *m/z* 902.44 (*z* = 3) was fragmented by MS/MS (Fig. 7*E*). The fragmentation pattern was consistent with a peptidoglycan peptide dimer consisting of the cell wall tripeptide d-Glu-mDAP-d-Ala cross-linked to the cell wall dipeptide d-Glu-mDAP that is branched to the C-terminal end of CD2831_R-HA_ peptide (Table 6). Furthermore, MS/MS analysis revealed a mDAP-mDAP cross-link generated by dl-transpeptidation because the daughter ion at *m/z* 873.04 (*z* = 3) corresponded to the loss of one alanine residue (−89.05) from the C-terminal end of the peptide stem. This feature is in complete agreement with the structure of the peptidoglycan of *C. difficile* as ∼75% of the cross-links have been shown to be generated by dl-transpeptidation (34).

**TABLE 6:** **Proposed structures of fragment ions from MS/MS collision-induced dissociation of parental ion at *m/z* 902.44 (*z* = 3)** See Fig. 7*E.*

Ion formed	*m/z*		Proposed structure
	Calculated	Observed	
[M + 2H]^2+^	575.85	575.86	mDAP- (PPVPPKT-)mDAP-d-Ala
[M + 2H]^2+^	640.37	640.39	d-Glu-mDAP- (PPVPPKT-)mDAP-d-Ala
[M + 3H]^3+^	816.42	816.86	mDAP- (YPYDVPDYAVNPPVPPKT-)mDAP-d-Ala
[M + 3H]^3+^	859.43	859.63	(d-Glu-(YPYDVPDYAVNPPVPPKT-)mDAP)-mDAP-d-Ala
[M + 3H]^3+^	872.76	873.04	d-Glu- (d-Glu- (YPYDVPDYAVNPPVPPKT-)mDAP)-mDAP
[M + 2H]^2+^	970.01	970.16	(d-Glu-(PDYAVNPPVPPKT-)mDAP)-mDAP-d-Ala
M + H^+^	1084.46	1084.21	YPYDVPDYA
[M + 2H]^2+^	1158.08	1158.16	d-Glu- (YPYDVPDYAVNPPVPPKT-)mDAP
[M + H]^+^	1183.53	1183.41	YPYDVPDYAV
[M + H]^+^	1297.57	1297.37	YPYDVPDYAVN
[M + H]^+^	1590.75	1590.64	YPYDVPDYAVNPPV

Because the peptidoglycan hydrolase used in this study is predicted to have *N*-acetylmuramoyl-l-amidase activity, the expected cleavage was between the muramic acid of the glycan chain and the l-alanine residue of the peptide chain stem. Thus, we expected to find an alanine residue at the N-terminal extremity of the generated peptidoglycan peptides. Yet, glutamate was found to be the N-terminal amino acid of the cell wall peptide side chain in all the elucidated structures. However, in our study the cell wall digestion was performed on intact bacteria rather than purified peptidoglycan. Thus, cleavage of the l-Ala-d-Glu cell wall peptide may be a feature of *C. difficile* endogenous peptidoglycan hydrolases able to digest peptidoglycan after its primary cleavage by the putative amidase. An alternative explanation could be related to the catalytic specificity of CD27L. Specificity of this endolysin has been deduced from its crystal structure and sequence comparisons, but there is no experimental evidence on its specificity (22). Therefore, CD27L might be an endopeptidase cleaving between l-alanine and d-glutamine residues.

These results demonstrate unambiguously that anchoring of CD2831 to the cell surface requires the cleavage of the polypeptide chain between the threonine and glycine residues of the predicted PPKTG-sorting motif by SrtB and that the resulting C-terminal threonine residue is then covalently anchored to the side chain amino group of mDAP, the third amino acid of the peptidoglycan peptide stem.

##### Expression and Secretion of CD2831 Are Controlled by c-diGMP Levels

**FIGURE 8.**
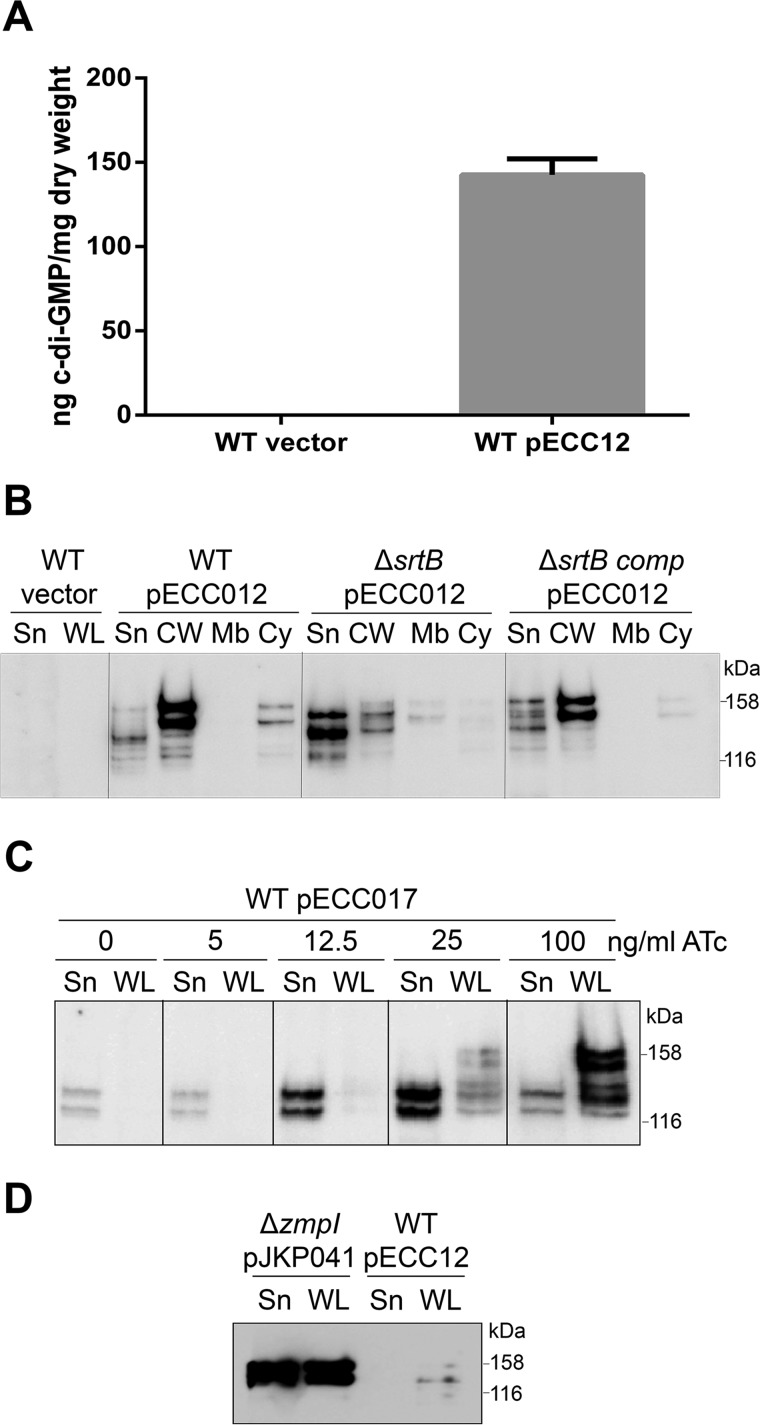
**Intracellular c-diGMP regulates the production and the subcellular localization of CD2831.** *A,* concentration of c-diGMP in the cytoplasm of the wild-type strain (*WT*) carrying an empty plasmid (vector) or pECC12 (constitutively expresses DccA) (*gray bar*) was quantified by LC-MS/MS. Cyclic dinucleotide concentrations are presented as nanograms of c-diGMP/mg of bacterial dry weight, and the average and standard deviation of three values are plotted. *B,* supernatant (*Sn*), cell wall (*Cw*), membrane (*Mb*), and cytosolic (*Cy*) compartments of wild-type (*WT*), sortase mutant (Δ*srtB*), and complemented sortase (Δ*srtB_comp_*) strains carrying pECC12 (constitutively expresses DccA) were analyzed by Western blotting with anti-CD2831 antibodies. Supernatant and whole cell lysate (*WL*) fractions of wild-type strain carrying an empty plasmid (vector) were also analyzed as negative controls. Proteins were separated on 6% SDS-polyacrylamide gels. *C,* supernatant (*Sn*) and whole cell lysate (*WL*) fractions of wild-type strain (*WT*) carrying an empty plasmid (vector) or pECC17 (expresses DccA in the presence of ATc) and induced with the indicated concentrations of ATc were immunoblotted with anti-CD2831 antibodies. Proteins were separated on 6% SDS-polyacrylamide gels. *D,* supernatant (*Sn*) and whole cell lysate (*WL*) fractions of *zmpI* mutant strain (Δ*zmpI*), carrying pJKP041 (expresses CD2831 in the presence of ATc) and cultivated in the presence of 100 ng/ml ATc, and of wild-type strain (*WT*), carrying pECC12 (constitutively expresses DccA), were immunoblotted with anti-CD2831 antibodies.

Transcription of *CD2831* is controlled by a type II c-diGMP riboswitch (Cdi2_3) and is increased in the presence of high levels of c-diGMP (20). Interestingly, expression of the adjacent gene *CD2830* encoding ZmpI protease is negatively regulated by c-diGMP through a type I riboswitch (Cdi1_12) (20). Because *zmpI* and *CD2831* are inversely regulated by c-diGMP, we investigated whether CD2831 is anchored to the cell surface in the presence of high levels of c-diGMP. The *dccA* gene encoding a c-diGMP synthetase was cloned in a plasmid under the control of the constitutive *P_cwp2_* promoter, leading to pECC12. Measurement of the intracellular c-diGMP concentration by LC-MS/MS confirmed the presence of high levels of c-diGMP in the wild-type strain containing pECC12 (142.57 ± 9.57 ng/mg bacterial dry weight), whereas c-diGMP was undetectable in the strain carrying an empty vector (Fig. 8*A*). Western blot analysis of subcellular fractions revealed that expression of CD2831 was induced in the presence of a high level of c-diGMP, as the protein could be detected without using any expression in *trans* (Fig. 8*B*). Moreover, CD2831 was almost exclusively detected in the cell wall fraction as the two predominant polypeptides of ∼140 and ∼155 kDa, suggesting that production of the ZmpI protease was abolished in these conditions. CD2831 could not be detected in the supernatant or the whole cell protein extract from the wild-type strain carrying an empty plasmid. When pECC12 was introduced into 630 Δ*srtB*, CD2831 could be detected but was mostly found in the culture supernatant, consistent with the requirement of the sortase to anchor CD2831 to the cell wall. To complement the Δ*srtB* mutant, the *srtB* gene was restored to its native locus using allelic replacement. To distinguish the complemented strain from the wild-type strain, the original TAA stop codon of *srtB* was replaced with a TAG stop codon in the complemented *srtB* strain. Plasmid pECC12 was transferred into the resulting strain, *srtB*_comp_, and wild-type CD2831 localization was restored in this strain (Fig. 8*B*).

To be able to modulate more precisely the c-diGMP levels in *C. difficile*, plasmid pECC17, expressing DccA under control of the *P_tet_*-inducible promoter, was constructed and introduced into the wild-type strain. Expression of *dccA* was then induced by a range of ATc concentrations, and CD2831 levels were determined by immunoblotting (Fig. 8*C*). As expected, CD2831 levels observed were directly dependent on the dose of ATc added. Only faint bands could be observed upon induction with 5 ng/ml ATc, and strong bands were present upon induction with 25 or 100 ng/ml ATc. Furthermore, when cultures were induced with 12.5 ng/ml ATc or below, CD2831 was found exclusively in the supernatant fraction and at a size suggesting its cleavage by ZmpI. In contrast, upon induction with 100 ng/ml ATc, a large proportion of CD2831 is found at a higher mass in the whole cell fraction suggesting that the c-diGMP levels are sufficient to strongly deplete expression of ZmpI. An intermediate state was also observed when strain was induced with 25 ng/ml ATc, with CD2831 found both in the supernatant and the whole cell lysate fractions. These results indicate that the ZmpI-mediated switch from surface exposure of CD2831 to its release into the extracellular medium release is tightly controlled because a threshold c-diGMP concentration appears to be required to activate or repress the ZmpI expression.

**FIGURE 9.**
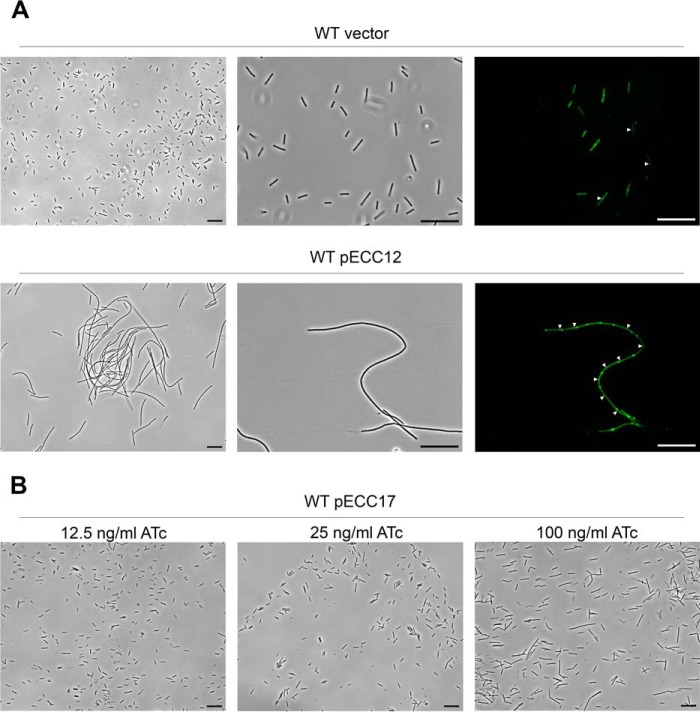
**Elevated c-diGMP levels induce formation of long chain of cells in *C. difficile* 630.** *A,* phase contrast (*left* and *center panels*) and fluorescence (*right panel*) microscopy images *C. difficile* wild-type (*WT*) strain carrying an empty plasmid (vector) or pECC12 (constitutively expresses DccA). For the fluorescence microscopy, membranes were stained with MitoTracker Green. *White triangles* indicate positions of septa. *Scale bars,* 20 μm. *B,* phase contrast microscopy views of *C. difficile* wild-type (*WT*) strain carrying pECC17 (expresses DccA in the presence of ATc). Expression of DccA from pECC17 was induced with the indicated concentrations of ATc. *Black bars,* 20 μm.

Immunofluorescence microscopy analysis was performed to detect CD2831. In the wild-type strain carrying pECC12 (constitutive expression of *dccA*) or pECC17 and grown in the presence of 100 ng/ml ATc (inducible expression of *dccA*), fluorescence was not detected (data not shown). Phase contrast microscopy revealed that cells carrying pECC12 grew as chains compared with the wild-type strain carrying an empty vector (Fig. 9*A*). These chains contained incomplete cell septa as visualized by staining with MitoTracker Green, suggesting that increased intracellular levels of c-diGMP result in aberrant cell division. Chains of cells were also observed in the strain carrying pECC17 upon induction with 100 ng/ml ATc, but to a much lesser extent (Fig. 9*B*). Indeed, in the strain carrying pECC17, chain length was found to correlate to the inducer concentration. Thus in *C. difficile* 630, formation of long chains of cells is likely the direct consequence of the elevation of c-diGMP levels. The absence of CD2831 immunolabeling at the surface of cells expressing high levels of c-diGMP might be related to the formation of chains or to insufficient levels of surface-localized CD2831. To explore this second hypothesis, the levels of CD2831 in strains 630 (pECC12) and 630 Δ*zmp1* (pJKP041) were compared. The levels of CD2831 were extremely low in 630 (pECC12) compared with 630 Δ*zmp1* (pJKP041), after induction with ATc, and were therefore probably too low to be detected by immunofluorescence (Fig. 8*D*).

##### CD3246 Is a Second SrtB Substrate Released by the ZmpI Protease

**FIGURE 10.**
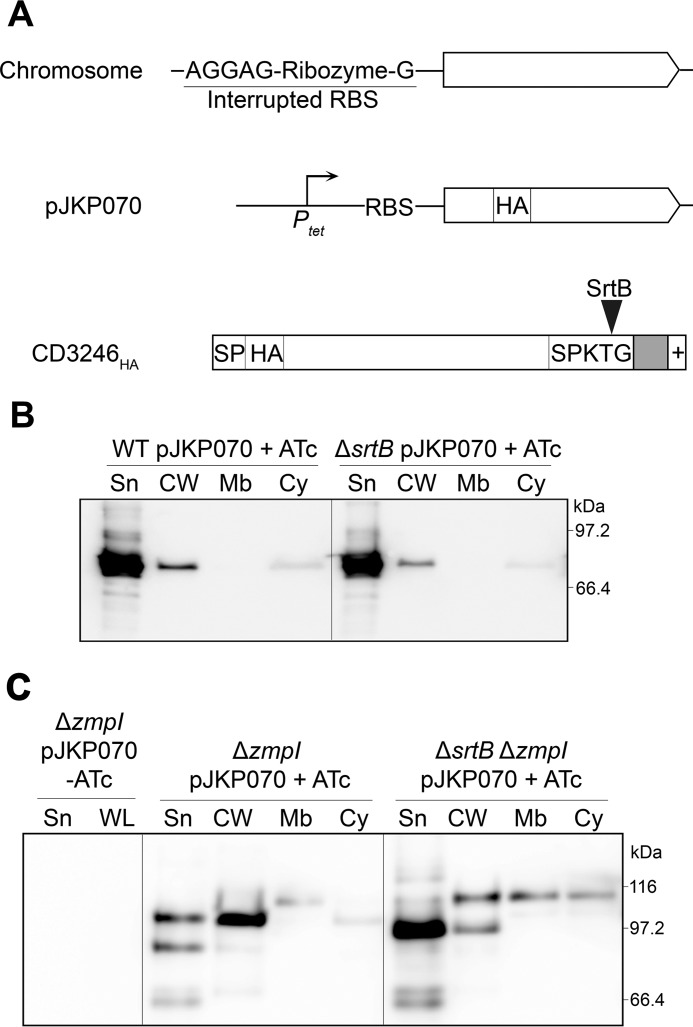
**CD3246_HA_ is anchored to the cell wall by SrtB and is released into the extracellular medium through its cleavage by ZmpI.** *A,* schematic representation of chromosomal *CD3246* showing its ribozyme-interrupted RBS and of CD3246_HA_ encoded by pJKP070 under control of the *P_tet_* inducible promoter and a functional RBS. The signal peptide (*SP*), the HA tag (*HA*), and the sorting signal composed of the SPKTG motif, the hydrophobic domain (in *gray*), and the positively charged tail (+) are represented. The SPKTG sorting motif is cleaved by *srtB* between the threonine (*T*) and the glycine (*G*) residues. *B,* supernatant (*Sn*), cell wall (*CW*), membrane (*Mb*), and cytosolic (*Cy*) compartment of wild-type (*WT*) and sortase mutant (Δ*srtB*) strains carrying pJKP070 (expresses CD3246_HA_ in the presence of ATc) and cultivated in the presence of 100 ng/ml ATc (+*ATc*) were analyzed by Western blotting with anti-HA monoclonal antibodies. Proteins were separated on 12% SDS-polyacrylamide gels. *C,* supernatant (*Sn*), cell wall (*CW*), membrane (*Mb*), and cytosolic (*Cy*) compartment of *zmpI* mutant (Δ*zmpI*) and *srtB*/*zmpI* double mutant (Δ*srtB*Δ*zmpI*) strains, carrying pJKP070 (expresses CD3246_HA_ in the presence of ATc) and cultivated in the presence of 100 ng/ml ATc (+*ATc*), were analyzed by Western blotting with anti-HA monoclonal antibodies. Supernatant (*Sn*) and whole cell lysate (*WL*) fractions of Δ*zmpI* pJKP070 strain cultivated in the absence of ATc ([*minus]ATc*) were also analyzed as negative controls.

CD3246, identified as another putative SrtB substrate, harbors a SPKTG sorting motif and is annotated as a putative adhesin. CD3246 is not conserved across all *C. difficile* lineages and is notably absent from ribotype 027 strains. CD3246 shares similarities with CD2831. First, a c-diGMP-II riboswitch (Cdi2_1) is present upstream of CD3246 (20). This riboswitch controls transcription and also initiation of translation through the splicing of an allosteric self-splicing ribozyme, located between the riboswitch and the CD3246-coding sequence (Fig. 10*A*) (35, 36). Second, CD3246 contains seven consecutive ZmpI cleavage sites gathered in the C-terminal extremity of the protein (30). We therefore investigated the subcellular localization of CD3246 to determine whether surface attachment of CD3246 is controlled by the same mechanism as that of CD2831. We constructed a derivative of CD3246 with an HA tag inserted five residues downstream of the signal peptide. A functional RBS was also inserted upstream of the CD3246-coding sequence because the native CD3246 RBS is interrupted by the ribozyme (Fig. 10*A*). This construct was then placed under the control of *P_tet_*, yielding pJKP070, and introduced into *C. difficile* 630 and Δ*srtB* strains. Upon induction, anti-HA antibodies detected the majority of the CD3246_HA_ in the culture supernatant of 630 (pJKP070) and a small proportion in the cell wall fraction. A similar profile was obtained with the Δ*srtB* (pJKP070) strain grown in the presence of ATc, indicating that the low level of cell wall associated CD3246_HA_ is not mediated by SrtB (Fig. 10*B*). Plasmid pJKP070 was then introduced into the Δ*zmpI* mutant. As expected, subcellular localization of CD3246_HA_ was affected by the *zmpI* deletion with the majority of the protein now found associated to the cell wall (Fig. 10*C*). Also, the apparent molecular weight of CD3246_HA_ was significantly larger in the absence of ZmpI, in agreement with the proteolytic activity of ZmpI on CD3246 (Fig. 10, *B* and *C*).

When expressed in the Δ*zmpI*Δ*srtB* double mutant, the majority of CD3246_HA_ was secreted (Fig. 10*C*). The thick band detected in the supernatant fraction had an apparent molecular weight slightly lower than that previously observed in the cell wall of 630 Δ*zmpI* (pJKP070), suggesting proteolytic degradation of CD3246_HA_ when it is not associated with the cell wall. Moreover, a faint band with a mass slightly below 116 kDa could be observed in the cell wall, membrane, and cytoplasm fractions. This band might correspond to the full-length CD3246_HA_ protein, as the C-terminal sorting signal cannot be cleaved in absence of SrtB. The supernatant fraction and whole cell extract from Δ*zmpI* (pJKP070) grown in the absence of inducer were also used as negative controls to confirm the specificity of anti-HA antibodies against CD3246_HA_. These data demonstrate that SrtB is required for CD3246_HA_ anchoring to the cell wall and strongly suggest that the surface exposure of the SrtB substrate CD3246 is controlled by c-diGMP levels through its ZmpI-mediated cleavage.

##### Physiological Studies of the Strain Devoid of the SrtB Sortase in Presence of Low or High Levels of c-diGMP

As our data indicate the cell wall association of at least two SrtB substrates is dependent on the intracellular c-diGMP levels, we investigated the physiological role of SrtB in *C. difficile* in the presence of c-diGMP. Wild-type and Δ*srtB* strains carrying pECC17 or a control vector exhibited similar growth rates in BHIS medium regardless of induction with ATc (data not shown).

Two phenotypes have been associated with varying levels of c-diGMP in *C. difficile-*biofilm formation and cell aggregation (20, 27). However, under the conditions used, neither the wild-type strain carrying pECC17 nor the empty vector was able to form a significant biofilm, limiting any comparison with the Δ*srtB* mutant strains (data not shown). Differing accounts of biofilm formation in *C. difficile* have been described (20, 37, 38) as follows: specifically low or negligible levels of biofilm formation in strain 630 but higher levels in strain R20291.

Cell aggregation was compared in liquid cultures of wild-type and Δ*srtB* strains carrying pECC17. In agreement with the previous reports (27, 37), aggregation was observed for the wild-type strain carrying pECC17 upon induction of *dccA* but not in the wild-type strain with a control vector. Deletion of *srtB* had no effect on cell aggregation, and upon ATc induction, a similar degree of aggregation was observed in 630 or the Δ*srtB* strain carrying pECC17 (data not shown). These results suggest that SrtB does not contribute to c-diGMP-mediated aggregation.

We found that high c-diGMP levels led to the formation of long chains of cells (see above). Phase contrast microscopy of Δ*srtB* carrying pECC12 or pECC17 grown in the presence of ATc did not reveal an involvement of sortase, as chain length was similar in all strains grown under identical conditions (data not shown).

Sporulation rate was also compared between these strains in the presence of 100 ng/ml ATc. Although the total cell count was identical in all four strains, strains carrying pECC17 produced many more spores (6–11 spores per 10^5^ cells) than the strain carrying an empty vector (2.2–4.5 per 10^6^ cells) after 24 h of growth. However, the sortase deletion had no significant effect on the sporulation rate. After 72 h of growth, the total cell count was similar, and sporulation rate was no longer significantly different (1.5–2.7 spores per 10^2^ cells) between the four strains (Table 7). These data indicate that SrtB substrates do not play a prominent role in sporulation or germination.

**TABLE 7:** **Total cell counts and sporulation rates for wild-type and Δ*srtB* mutant strains carrying a control vector or pECC17 (expresses DccA in the presence of ATc) in the presence of 100 ng/ml ATc** Values are means of results from four independent experiments.

Culture time(h)	Unit	Mean value for the strain (±S.D.)*^a^*			
		WT vector	Δ*srtB* vector	WT pECC17	Δ*srtB* pECC17
24	cfu/ml (×10^7^)	11.59 (2.6)	13.77 (1.27)	10.17 (2.35)	11.94 (3.21)
	% spores (×10^−3^)*^a^*	0.45 (0.44)	0.22 (0.068)	11.01 (9.75)	6.34 (5.65)
72	cfu/ml (×10^7^)	0.47 (0.31)	0.45 (0.28)	0.69 (0.62)	0.31 (0.08)
	% spores (×10^0^)*^b^*	2.74 (2.76)	2.14 (1.58)	1.81 (1.91)	1.47 (0.42)

*^a^* Statistical significance for spore formation differences between strains WT vector and WT pECC17 was evaluated by a Wilcoxon test (*p* = 0.028).

*^b^* Statistical significance for spore formation differences between strains WT vector and WT pECC17 was evaluated by a Wilcoxon test (*p* = 0.89).

## Discussion

Cyclic diGMP is a second messenger in bacterial systems and a key feature in the control of critical lifestyle choices, such as the transition between planktonic and biofilm growth. Elevated levels of c-diGMP typically promote sessile lifestyles such as biofilm formation; in contrast, low levels of c-diGMP are associated with motility (39–41). *C. difficile* encodes 18 predicted diguanylate cyclases and 19 predicted phosphodiesterases, many of which have confirmed enzymatic activity (42, 43), revealing the importance of c-diGMP signaling in this human pathogen.

We show that c-diGMP controls surface attachment of the putative adhesin CD2831 by controlling its expression and mediating its cleavage and release from the cell wall by the zinc metalloprotease ZmpI. ZmpI (CD2830) and CD2831 are encoded on adjacent genes on the chromosome. Upstream of *zmpI* is the c-diGMP-I riboswitch Cdi1_12 and upstream of *CD2831* is the c-diGMP-II riboswitch Cdi2_3. Elevated levels of c-diGMP repress transcription of *zmpI* and increase transcription of *CD2831* (20).

We show that c-diGMP-mediated regulation directly impacts the levels of CD2831 and presumably ZmpI proteins. CD2831 can be detected only when cells are producing high levels of c-diGMP, where it is found in the cell wall. In cells producing low levels of c-diGMP, CD2831 is efficiently cleaved and released into the supernatant by ZmpI. This suggests the absence of ZmpI when intracellular elevated levels of c-diGMP are high.

The model emerging from this finding is that at low c-diGMP levels, a motile lifestyle is promoted. Expression of the adhesin CD2831 is repressed, and residual low levels of surface-localized protein will be shaved from the cell surface and released into the supernatant through its cleavage by ZmpI, which is produced under these conditions. In contrast, at elevated levels of c-diGMP, a sessile lifestyle is promoted. Expression of CD2831 is induced and, in the absence of ZmpI activity, anchored at the cell surface by sortase. Thus, protease ZmpI might play a key role in the transition from a motile to a sessile lifestyle or additionally from a sessile to a motile lifestyle.

In *C. difficile*, c-diGMP has been reported to inhibit swimming motility and toxin production and to promote biofilm formation, type IV pili biogenesis, and cell aggregation (20, 27, 37, 44). Hence, the increased levels of c-diGMP promote a sedentary lifestyle. We envisage that *in vivo*, once sedentary, the bacteria may use the adhesins CD2831 and CD3246 to interact with extracellular matrix proteins such as collagens and/or fibronectin to reduce the possibility of movement and to commence biofilm formation. However, we were unable to detect efficient binding of these proteins to extracellular matrix components *in vitro*, suggesting perhaps that such interactions are highly specific for substrates or conditions found *in vivo*.

Numerous bacterial proteases function as virulence factors by facilitating infection of the host, and control of protease activity by mechanisms involving c-diGMP has already been reported. For example, in the obligatory intracellular bacterium *Ehrlichia chaffeensis,* c-diGMP regulates intracellular aggregation and release from host cells through stabilization of bacterial surface-exposed proteins by preventing their degradation by endogenous proteases (45, 46). In *Pseudomonas fluorescens,* a complex molecular mechanism regulating LapA proteolysis in a c-diGMP-dependent manner has been elegantly deciphered. When c-diGMP is present at low levels within cells, the N-terminal extremity of the surface-exposed adhesin LapA is cleaved by the periplasmic cysteine protease LapG, thus releasing LapA from the cell surface and leading to the loss of the ability to form biofilm. In contrast, when c-diGMP levels are elevated, the inner membrane-localized LapD protein sequesters LapG via direct protein-protein interactions, preventing proteolytic processing of LapA. Regulation of this direct protein-protein interaction is mediated by the binding of intracellular c-diGMP to LapD, which induces a conformational change of the LapD periplasmic domain (47, 48).

In a recent study, Purcell *et al.* (27) successfully detected c-diGMP in *C. difficile* 630 and reported its concentration to be low compared with other bacteria. As intracellular c-diGMP was undetectable in wild-type cells in our study, we assume that these low levels were below the limit of detection, *i.e.* 0.5 ng/mg dry weight of c-diGMP, under our experimental conditions. Elevated c-diGMP levels have been shown to cause aggregation of *C. difficile,* and scanning electron microscopy revealed the presence of fibrous networks gathering cells in large aggregates (27). In agreement with these data, we observed extensive cell aggregation when c-diGMP levels were high, but additionally we noticed the formation of long chains of cells in this condition. This phenotype is particularly marked when c-diGMP is produced at very high levels with expression of DccA under control of the constitutive *P_cwp2_* promoter. Absence of this phenotype in the study of Purcell *et al.* (27) might be attributable to the experimental conditions used by the two groups (*e.g.* different systems for DccA overexpression), possibly leading to higher c-diGMP levels in our work. Establishment of long chains of cells is likely a direct consequence of increased c-diGMP levels, but the mechanism involved remains to be determined. The phenotype is likely to be associated with a defect in cell separation and might therefore be due to the control of the expression or activity of one or several peptidoglycan hydrolases, as they are essential for septum formation and cell separation (49). Interestingly, *Streptococcus mutans*, a strain lacking the diguanylate cyclase Gcp and presumably producing low levels of c-diGMP, forms extremely long chains of cells (50). This opposite regulation highlights the fact that c-diGMP signaling can play a diverse role in different bacteria. Although beyond the scope of this study, it will be interesting to further investigate the relation between c-diGMP and cell separation defect in *C. difficile*.

We show functionality of SrtB in *C. difficile* and elucidate the first anchor structure for a *Clostridium* species. SrtB is required for anchoring CD2831 to the cell surface. SrtB cleaves the PPKTG motif of CD2831 between the threonine and glycine residues and tethers the carboxyl group of threonine to the amino group of DAP cross-bridges of peptidoglycan. *srtB* is the only intact sortase gene in *C. difficile*, and van Leeuwen and co-workers (30) speculated that SrtB might be the “housekeeping” sortase of *C. difficile* with an activity similar to class A sortases in other Gram-positive species. In agreement with this hypothesis is the close resemblance between the (S/P)P*X*TG motif recognized by *C. difficile* SrtB and the LP*X*TG motif recognized by class A sortases. Moreover, class B sortases are generally expressed in operons containing genes encoding their substrates, which is not the case in *C. difficile* (18).

Are the *C. difficile* SrtB substrates initially linked to the lipid II peptidoglycan precursors or directly incorporated into the mature peptidoglycan? In our study, immunofluorescence microscopy revealed that high expression levels of CD2831 in a strain lacking the protease ZmpI results in asymmetric localization of CD2831 to one cell pole. Similar observations have been reported for the SrtB substrate Hbp2 (SvpA) in *L. monocytogenes* (32). This species has both class A and B sortases, and a model for the spatial cell localization of their respective substrates has been proposed (14, 51). When the SrtA substrate InlA is produced at high levels, it accumulates at the septal region and is anchored to lipid II peptidoglycan precursors at the newly formed pole. At the next cell division, InlA is incorporated at the new pole leading to the symmetrical accumulation of InlA at both poles. In contrast, the SrtB substrate Hbp2 is thought to be anchored directly to the mature peptidoglycan. It therefore accumulates at old cell poles rather than at newly formed poles, leading to an asymmetrical distribution of Hbp2 at one cell pole only.

*C. difficile* is atypical among Gram-positive bacteria in processing a single active sortase. This may explain some of the unusual features of the role of SrtB. The sortase substrates found in *C. difficile* appear to have properties usually associated with class A sortases, *e.g.* collagen-binding proteins, as opposed to pili or other functional classes normally associated with sortase B enzymes. However, the apparent use of assembled peptidoglycan as a substrate and amino acid sequence homology points to a class B enzyme.

Further investigation into the role of SrtB substrates CD2831 and CD3246 in the physiology of *C. difficile* will be required to give a full insight into the importance of the link between two major bacterial processes as follows: the putative virulence factor cell wall attachment catalyzed by sortase enzymes and regulation of virulence by second messenger c-diGMP. Such studies could include detailed kinetics of colonization of the mouse enteric tissues using defined *srtB* mutants and sortase substrates.

## Author Contributions

J. P. and N. F. F. conceived and coordinated the study, which was initiated by H. A. S. J. P. performed the majority of the experiments. H. A. S. conceived several experiments, expressed and purified the recombinant catalytic domain of endolysin CD27L, and helped design the cell fractionation protocol. E. C. C. designed and constructed vectors for constitutive and conditional expression of DccA in *C. difficile*. L. F. D. and B. W. W. provided scientific insight into the design of experiments. L. Y. and J. S. C. performed the mass spectrometry to determine the anchor structure of CD2831. V. K. performed the mass spectrometry to measure the c-diGMP levels. J. P. and N. F. F. wrote the paper, and all authors reviewed the results and approved the final version of the manuscript.

## Supplementary Material

Supplemental Data

## Footnotes

5 The abbreviations used are:

mDAP: *meso-*diaminopimelic acid
ATc: anhydrotetracycline
c-diGMP: cyclic diGMP
RBS: ribosome-binding site
R-HA: arginyl-hemagglutinin.
