# Supplementary material for: Cyclic diGMP Regulates Production of Sortase Substrates of Clostridium difficile and Their Surface Exposure through ZmpI Protease-mediated Cleavage
Source: J Biol Chem. 2015 Aug 17;290(40):24453–69. doi: 10.1074/jbc.M115.665091 (PMC4591827; doi:10.1074/jbc.M115.665091)
Supplement: Supplemental Data [file supp_290_40_24453__index.html]

Cyclic-di-GMP regulates production of sortase substrates of Clostridium difficile and their surface exposure through ZmpI protease-mediated cleavage — Cyclic diGMP Regulates Production of Sortase Substrates of Clostridium difficile and Their Surface Exposure through ZmpI Protease-mediated Cleavage — c-diGMP Controls Surface Exposure of Sortase Substrates — Supplemental Data 

# Cyclic diGMP Regulates Production of Sortase Substrates of *Clostridium difficile* and Their Surface Exposure through ZmpI Protease-mediated Cleavage

## Supplemental Data

- Supplemental Table S1 (.pdf, 128 KB) - Supplemantal Table 1 detailing primers, plasmids ans strains
