# Supplementary material for: Cyclic diGMP Regulates Production of Sortase Substrates of Clostridium difficile and Their Surface Exposure through ZmpI Protease-mediated Cleavage
Source: J Biol Chem. 2015 Aug 17;290(40):24453–69. doi: 10.1074/jbc.M115.665091 (PMC4591827; doi:10.1074/jbc.M115.665091)
Supplement: Supplemental Data [file supp_M115.665091_jbc.M115.665091-1.pdf]

Cyclic-di-GMP regulates production of sortase substrates of *Clostridium difficile* and their surface exposure through ZmpI protease-mediated cleavage*

Johann Peltier[1], Helen A. Shaw[1], Edward C. Couchman[1], Lisa F. Dawson[2], Lu Yu[3], Jyoti S. Choudhary[3], Volkhard Kaever[4], Brendan W. Wren[2] and Neil F. Fairweather[1]

Supplemental material

Table S1. Strains, plasmids and oligonucleotides used for the present study.

Strain, plasmid or primer	Characteristic/description	Source/construction
**Strains**		
E. coli NEB5α	General cloning	New England Biolabs
E. coli Rosetta	Protein expression	Novagen
E. coli CA434	Conjugation donor	(1)
<u>*C. difficile* strains</u>		
630	Wild type Erm^R. Virulent and multidrug-resistant PCR ribotype 01	(2)
630Δ*erm*	Erythromycin-sensitive derivative of wild-type *C. difficile* 630	(3)
Δ*srtB*	630 isogenic *srtB* deletion mutant	This study
Δ*zmpI*	630 isogenic *zmpI* deletion mutant	This study
Δ*srtB*- Δ*zmpI*	630 isogenic double mutant with deletions in the aforementioned genes	This study
P_{tet}-*srtB*	Insertion of the Ptet promoter upstream *srtB* of the 630 strain by allele exchange	This study
Δ$srtB_{comp}$	Δ*srtB* complemented on the chromosome by allele exchange	This study
**Plasmids**		
pET28a	*E. coli* expression vector. Kan^R	Novagen
pUC19	*E. coli* cloning vector. Amp^R	New England Biolabs
pMTL-SC7215	*E. coli*-*C. difficile* shuttle vector for *codA*-mediated allele exchange mutagenesis. Tm^R	(4)
pRPF144	*E. coli*-*C. difficile* shuttle vector for protein expression. P_{cwp2}-*gusA*,Tm^R	(5)
pRPF185	*E. coli*-*C. difficile* shuttle vector for protein expression. P_{tet}-*gusA*, Tm^R	(5)
pHAS027	CD2831 (32-290)-His_6 in pET28a	This study
pHAS042	His_6-CD27L (1-179) in pET28a	This study
pJKP001	SrtB (27-225)-His_6 in pET28a	This study
pJKP019	pUC19 with construct for *srtB* deletion	This study
pJKP022	pMTL-SC7215 with construct for *srtB* deletion	This study

pJKP029	pMTL-SC7215 with construct for P_{tet}-*srtB* strain creation	This study
pJKP041	pRPF185 derivative carrying P_{tet}-*CD2831* for inducible CD2831 expression	This study
pJKP070	pRFP185 derivative carrying P_{tet}-*CD3392* with addition of a HA-tag encoding sequence into *CD3392* and a functional RBS upstream *CD3392* for inducible CD3392$_{HA}$ expression	This study
pJKP088	pMTL-SC7215 with construct for *zmpI* deletion	This study
pJKP095	pJKP041 with addition of R-HA-tag encoding sequence into *CD2831*	This study
pJKP096	pMTL-SC7215 with construct for *srtB* complementation	This study
pECC12	pRFP144 derivative carrying P_{cwp2}-*dccA* with addition of His-tag encoding sequence into 3'-extremity of dccA for constitutive expression of DccA.	This study
pECC17	pRFP185 derivative carrying P_{tet}-dccA with addition of His-tag encoding sequence into 3'-extremity of *dccA* for inducible expression of DccA.	This study

**Primer**	**Sequence (5' to 3')***	**Characteristics or use**
NF1614	GGGCCATGGATTCAGAATTAGGAGAGAATAGTCAGATTC	Amplify 5' extremity of CD2831 for pET28a cloning
NF1615	GGGCTCGAGTTCTATTTTTCCAGTAGTTTCAATATGTATTTTTG	Amplify 5' extremity of CD2831 for pET28a cloning
NF1968	ATATCCATGGAACTTACCAAATACAATCATGATACTA	Amplify *srtB* deleted in the N-terminal hydrophobic domain for pET28a cloning
NF1969	TGAGCTCGAGAATCAATCTACCATGAATCACCAT	Amplify *srtB* deleted in the N-terminal hydrophobic domain for pET28a cloning
NF2126	GATCGAGCTCGTATTTTATTTTGGAGAAATTAATATGTTTAAAG	Amplify *dccA* with the addition of a C-terminal His-tag encoding sequence for pRFP144 or pRFP185 cloning.
NF2155	GATAGAATTCCCATTTTTAATATCTGTACTAACTTATATTAC	Construction of pJKP019 for *srtB* deletion
NF2156	GATAGGATCCGAGAAGACCTGTCCTTAGTAC	Construction of pJKP019 for *srtB* deletion
NF2157	GCCAGAATGGTGATTCATGG	Construction of pJKP019 for *srtB*

		deletion
NF2158	CTGTAAAGTATTACTAATACTAGTATAATGT	Construction of pJKP019 for *srtB* deletion
NF2169	GGATTTCACATTTGCCGTTTTGTAAAC	Screen for insert into pMTL-SC7215
NF2170	GATCTTTTCTACGGGGTCTGAC	Screen for insert into pMTL-SC7215
NF2171	GCTACATCTTATATCATATCATATTATTTACATC	Screen for single and double crossover for *srtB* deletion
NF2172	CGGTCCCTATCCGTCGC	Screen for single and double crossover for *srtB* deletion
NF2199	GATCGGATCCTTAATGGTGATGGTGATGGTGCTCGAGATAATCATTTTTATCAAATTTTTCTTGTTTTTC	Amplify dccA with the addition of a C-terminal His-tag encoding sequence for pRFP144 or pRFP185 cloning.
NF2214	AAACTCCTTTTTGATAATCTCATGACC	Linearize pMTL-SC7215 by inverse PCR at the PmeI site
NF2215	AAACTTAGGGTAACAAAAAACACCG	Linearize pMTL-SC7215 by inverse PCR at the PmeI site
NF2254	gttttttgttaccctaagtttGTAATATTTACATTGTGCAAAGTTG	Construction of pJKP029 for creation of P_{tet}-*srtB* strain
NF2257	atgtagtttaGGAGCTCAGATCTGTTAAC	Construction of pJKP029 for creation of P_{tet}-*srtB* strain
NF2258	tgagctccTAAACTACATTTAACTCGAGGTG	Construction of pJKP029 for creation of P_{tet}-*srtB* strain
NF2259	gattatcaaaaaggagtttCAATCTACCATGAATCACCATTC	Construction of pJKP029 for creation of P_{tet}-*srtB* strain
NF2260	CACTCAAAACCTTCACTCCTTAAATC	Screen for single and double crossover forcreation of P_{tet}-*srtB* strain
NF2261	CAGTTTAAAATTATTGTTTTAAACTGTGTCTG	Screen for single and double crossover for creation of P_{tet}-*srtB* strain
NF2266	cagaattcgTATATCTATATTACACTATTTTTGAATAAATTTAAATTTTTAG	Construction of pJKP029 for creation of P_{tet}-*srtB* strain
NF2267	atatagatataCGAATTCTGCATCAAGCTAG	Construction of pJKP029 for creation of P_{tet}-*srtB* strain
NF2378	GATAGAGCTCAAAATGAAAGGAGCATTAGATTTATG	Amplify *CD3392* for pRFP185 cloning
NF2379	GATAGGATCCTTATGATTTCTTCATTTTACGGC	Amplify *CD3392* for pRFP185 cloning
NF2451	GGATCCTATAAGTTTTAATAAAACTTTAAATAG	Remove CD3392 coding sequence by inverse PCR
NF2501	GATAGAGCTCGGAAGATTAGATTATAAATTATAGGTAAC	Amplify *CD2831* for pRFP185 cloning
NF2502	GATAGGATCCTAATTTGTATTTTTATTTCTTCTTAATACGATAAG	Amplify *CD2831* for pRFP185 cloning
NF2726	TTCCAGATTATGCTGGTCAGA	Add a sequence encoding for HA

	CACCAGATGTAGA	tag into the CD3246 coding sequence by inverse PCR
NF2727	CATCATATGGATAATTTGATTGTTCTTCTCCATTAAGTATTG	Add a sequence encoding for HA tag into the CD3246 coding sequence by inverse PCR
NF2858	AAATCTAATGCTCCTTTCATTTTGAGC	Remove CD3392 coding sequence by inverse PCR
NF2860	gaaaggagcattagatttTTGAAAACAAAAATTAAAAAATCAAGTATAATC	Amplify *CD3246* for pRFP185 cloning
NF2861	attaaaacttataggatccTTAATTTCTTTTTATTTTACATTGATACGC	Amplify *CD3246* for pRFP185 cloning
NF2922	gttttttgttaccctaagtttCATACCATATTATTCCAATATGCT	Construction of pJKP088 for *zmpI* deletion
NF2923	atcaatattcCAAACTTATAGCTTTTTGCAAAATTTAG	Construction of pJKP088 for *zmpI* deletion
NF2924	tataagtttgGAATATTGATATTATAGCTATTAATAATTTTTTACTTG	Construction of pJKP088 for *zmpI* deletion
NF2925	gattatcaaaaaggagtttGGGGAGACTATACGAAATG	Construction of pJKP088 for *zmpI* deletion
NF2926	CCTGTGCAAAGTTGGGTTAC	Screen for single and double crossover for *zmpI* deletion
NF2956	CTTTACCAGCATTTGAAAAATTAGCC	Screen for single and double crossover for *zmpI* deletion
NF2958	CAGATTATGCTGTAAATCCACCAGTACCACC	Add a sequence encoding for the R-HA tag into the CD2831 encoding gene of pJKP041 by inverse PCR
NF2959	GAACATCATATGGATACCGCAAAGTATCATCTTTAACTTTATTATTTTTTTC	Add a sequence encoding for the R-HA tag into the CD2831 encoding gene of pJKP041 by inverse PCR
NF2966	gttttttgttaccctaagtttCGTCTTCTTCTCTCTGATAG	Construction of pJKP096 for complementation of *srtB* mutant
NF2967	tcaatctacCATGAATCACCATTCTGGC	Construction of pJKP096 for complementation of *srtB* mutant
NF2968	tgattcatgGTAGATTGATTTAGAAACTCACAGACAC	Construction of pJKP096 for complementation of *srtB* mutant
NF2969	gattatcaaaaaggagtttCGGAAAGACCCCATGGAG	Construction of pJKP096 for complementation of *srtB* mutant
NF2970	GAAGGTCTTTATATAGGTTTAAAACC	Screen for single and double crossover for complementation of *srtB* mutant
NF2971	CGGATACCTGCGACAGG	Screen for single and double crossover for complementation of *srtB* mutant

*Underlined bases indicate engineered restriction sites; lowercase bases indicate overlapping sequences

REFERENCES

1. Purdy, D., O'Keeffe, T. A., Elmore, M., Herbert, M., McLeod, A., Bokori-Brown, M., Ostrowski, A., and Minton, N. P. (2002) Conjugative transfer of clostridial shuttle vectors from *Escherichia coli* to *Clostridium difficile* through circumvention of the restriction barrier. *Mol Microbiol* **46**, 439-452

2. Sebaihia, M., Wren, B. W., Mullany, P., Fairweather, N. F., Minton, N., Stabler, R., Thomson, N. R., Roberts, A. P., Cerdeno-Tarraga, A. M., Wang, H., Holden, M. T., Wright, A., Churcher, C., Quail, M. A., Baker, S., Bason, N., Brooks, K., Chillingworth, T., Cronin, A., Davis, P., Dowd, L., Fraser, A., Feltwell, T., Hance, Z., Holroyd, S., Jagels, K., Moule, S., Mungall, K., Price, C., Rabbinowitsch, E., Sharp, S., Simmonds, M., Stevens, K., Unwin, L., Whithead, S., Dupuy, B., Dougan, G., Barrell, B., and Parkhill, J. (2006) The multidrug-resistant human pathogen *Clostridium difficile* has a highly mobile, mosaic genome. *Nat Genet* **38**, 779-786

3. Hussain, H. A., Roberts, A. P., and Mullany, P. (2005) Generation of an erythromycin-sensitive derivative of *Clostridium difficile* strain 630 (630Δ*erm*) and demonstration that the conjugative transposon Tn916E enters the genome of this strain at multiple sites. *J Med Microbiol* **54**, 137-141

4. Cartman, S. T., Kelly, M. L., Heeg, D., Heap, J. T., and Minton, N. P. (2012) Precise Manipulation of the *Clostridium difficile* Chromosome Reveals a Lack of Association Between tcdC Genotype and Toxin Production. *Appl Environ Microbiol* **78**, 4683-4690

5. Fagan, R. P., and Fairweather, N. F. (2011) *Clostridium difficile* has two parallel and essential Sec secretion systems. *J Biol Chem* **286**, 27483-27493
